# Neurodevelopmental, cognitive, behavioural and mental health impairments following childhood malnutrition: a systematic review

**DOI:** 10.1136/bmjgh-2022-009330

**Published:** 2022-07-06

**Authors:** Amir Kirolos, Magdalena Goyheneix, Mike Kalmus Eliasz, Mphatso Chisala, Samantha Lissauer, Melissa Gladstone, Marko Kerac

**Affiliations:** 1 Department of Women's and Children's Health, Institute of Life Course and Medical Sciences, Faculty of Health and Life Sciences, University of Liverpool, Liverpool, UK; 2 Malawi-Liverpool-Wellcome Trust Clinical Research Programme, Blantyre, Blantyre, Malawi; 3 Fundación ACNUR Argentina (Agencia de la ONU para los Refugiados, UNHCR), Buenos Aires, Argentina; 4 Malawi Epidemiology and Intervention Research Unit, Lilongwe/Karonga, Malawi; 5 Institute of Infection, Veterinary & Ecological Sciences, University of Liverpool, Liverpool, UK; 6 Centre for Maternal, Child, Adolescent & Reproductive Health (MARCH), London School of Hygiene & Tropical Medicine, London, UK; 7 Department of Population Health, London School of Hygiene & Tropical Medicine, London, UK

**Keywords:** Nutrition, Public Health, Nutritional and metabolic disorders, Systematic review, Mental Health & Psychiatry

## Abstract

**Background:**

Severe childhood malnutrition impairs growth and development short-term, but current understanding of long-term outcomes is limited. We aimed to identify studies assessing neurodevelopmental, cognitive, behavioural and mental health outcomes following childhood malnutrition.

**Methods:**

We systematically searched MEDLINE, EMBASE, Global Health and PsycINFO for studies assessing these outcomes in those exposed to childhood malnutrition in low-income and middle-income settings. We included studies assessing undernutrition measured by low mid-upper arm circumference, weight-for-height, weight-for-age or nutritional oedema. We used guidelines for synthesis of results without meta-analysis to analyse three outcome areas: neurodevelopment, cognition/academic achievement, behaviour/mental health.

**Results:**

We identified 30 studies, including some long-term cohorts reporting outcomes through to adulthood. There is strong evidence that malnutrition in childhood negatively impacts neurodevelopment based on high-quality studies using validated neurodevelopmental assessment tools. There is also strong evidence that malnutrition impairs academic achievement with agreement across seven studies investigating this outcome. Eight of 11 studies showed an association between childhood malnutrition and impaired cognition. This moderate evidence is limited by some studies failing to measure important confounders such as socioeconomic status. Five of 7 studies found a difference in behavioural assessment scores in those exposed to childhood malnutrition compared with controls but this moderate evidence is similarly limited by unmeasured confounders. Mental health impacts were difficult to ascertain due to few studies with mixed results.

**Conclusions:**

Childhood malnutrition is associated with impaired neurodevelopment, academic achievement, cognition and behavioural problems but evidence regarding possible mental health impacts is inconclusive. Future research should explore the interplay of childhood and later-life adversities on these outcomes. While evidence on improving nutritional and clinical therapies to reduce long-term risks is also needed, preventing and eliminating child malnutrition is likely to be the best way of preventing long-term neurocognitive harms.

**PROSPERO registration number:**

CRD42021260498.

**WHAT IS ALREADY KNOWN ON THIS TOPIC**

- High mortality risk and impaired growth are well-recognised short-term risks of childhood malnutrition.
- While there is increasing appreciation of longer-term risks for survivors, notably adult cardiometabolic non-communicable disease, other longer-term risks have been poorly described.

**WHAT THIS STUDY ADDS**

- There is strong evidence that malnutrition impairs neurodevelopment and academic achievement in childhood which has significant implications for future well-being and prospects of those affected.
- Childhood malnutrition is associated with impaired cognition and behavioural problems with evidence of effects through to adolescence and adulthood but the effect of nutritional treatment and interplay with childhood adversity, coexisting illness such as HIV and environmental factors in influencing these outcomes is unclear.

**HOW THIS STUDY MIGHT AFFECT RESEARCH, PRACTICE AND/OR POLICY**

- Study findings imply that there are likely to be long-term effects of childhood malnutrition on cognition and well-being lasting through adolescence and adulthood.
- Long-term needs of malnutrition survivors need to be carefully considered in treatment programmes. Further research is needed on the effects of nutritional therapy, adversity and environmental factors to tailor future interventions, particularly with regard to mental health which has been little researched.

## Introduction

Severe childhood malnutrition is widespread and has a high disease burden concentrated in low-income and middle-income settings.^1^ To date, most malnutrition policies and treatment programmes have focused on short-term risks, notably infections and death.^2 3^ There is however growing evidence of long-term risks for malnutrition survivors, including that of later-life non-communicable disease.^4^ The prevalence of malnutrition has decreased in recent years due to concerted global efforts.^1^ However, there is a risk of resurgence and perpetuation of childhood malnutrition due to climate change, conflict and food insecurity in many settings with fragile food supply chains.^5^

Understanding long-term outcomes following child malnutrition is especially important because improved treatment has thankfully resulted in more children with malnutrition surviving into adolescence and adulthood.^3 6^ Most previous research and programmatic investment have focused on child mortality rather than thriving and long-term outcomes. Fewer studies explore long-term impacts of malnutrition on cognitive, behavioural and mental health outcomes in survivors. Improved understanding of these outcomes can inform disease burden estimates, support ongoing investment and inform follow-up care for children with malnutrition.

Causal pathways linking malnutrition with neurodevelopment, cognition, behaviour and mental health are complex. Previous studies have found children admitted to hospital with severe malnutrition often have severe developmental delays with significant implications for ongoing development, well-being and potential future capital.^7^ The interaction with HIV exposure in settings where both malnutrition and HIV are highly prevalent is also important given that both are associated, and both can independently affect early childhood development. Quantifying the association between malnutrition and neurodevelopment is complicated by the fact that children with neurodisability are inherently at higher risk of becoming malnourished, potentially due to factors such as poor feeding or differing treatment within family groups with food scarcity.^8^ Other factors may mediate outcomes such as socioeconomic adversity, risk of infectious disease, parental engagement and school attendance which have been found to influence early childhood development and are often associated with risk of developing severe malnutrition.^9^ These mediating factors also explain in part the difficulty in predicting developmental trajectories after an episode of severe malnutrition and why studies investigating outcomes are significantly influenced by potential confounders affecting the internal validity of results.^10^

A 1995 review found school-age children who suffered from early childhood malnutrition generally had poorer IQ levels, cognitive function, school achievement and greater behavioural problems than matched controls and, to a lesser extent, siblings.^11^ Despite these associations, previous evidence of direct causal relationships is limited due to a lack of long-term follow-up studies, retrospective study designs and few studies having investigated behaviour and mental health. With this lack of up-to-date evidence on an increasingly important topic we aimed to identify studies reporting neurodevelopmental, cognitive, behavioural and mental health outcomes for children exposed to malnutrition in childhood.

## Methods

We searched MEDLINE, EMBASE, Global Health and PsycINFO for studies published between 1 January 1995 and 6 January 2022 using Preferred Reporting Items for Systematic Reviews and Meta-Analyses guidelines.^12^ Detailed search strategies are included in online supplemental appendix 1.

10.1136/bmjgh-2022-009330.supp1 Supplementary data

### Selection criteria

We included studies from low-income and middle-income countries reporting neurodevelopmental, cognitive, school/academic achievement, mental health or behavioural outcomes in children under five exposed to malnutrition compared with children without malnutrition (we did not exclude studies that included small numbers of children between the ages of 5 and 6 years). We defined childhood malnutrition as undernutrition using standard definitions (definitions which are commonly measured in research and often related to severe adverse outcomes)^13^: moderate or severe wasting defined by low weight for height or low mid-upper arm circumference; presence of nutritional oedema; low weight-for-age as per older definitions of severe malnutrition. Despite the overlap between those suffering from severe undernutrition and those with micronutrient deficiencies and chronic malnutrition, we focus on acute undernutrition in early childhood in this review. We, therefore, did not include studies focusing solely on stunting (low height-for-age), chronic malnutrition or micronutrient deficiencies which often have their own dedicated literature.^14 15^

We included cross-sectional, cohort, case-control and controlled trial study designs which had a comparator group not exposed to childhood malnutrition. We excluded conference papers and reviews which did not include original data. We excluded studies which failed to define how malnutrition, child neurodevelopment, cognition, school/academic achievement, mental health or behaviour were measured. We also excluded studies looking at specific sub-populations of children (eg, only children with a specific medical condition). No language restrictions were placed on studies.

### Literature search

Titles and abstracts were screened by two independent reviewers (two of AK, MGo, MKE and MC). The full texts of titles and abstracts chosen by any reviewer were then independently reviewed by two reviewers (two of AK, MGo, MKE and MC) against our selection criteria. We also screened reference lists of included studies. Data extraction of included studies was done by one of AK or MGo into a standard template (including study characteristics, assessment tool, malnutrition definition, sample size, results in cases and controls, reported effect sizes, results of statistical significance tests, results of analyses adjusted for confounding variables). Data extraction was subsequently checked by a second separate reviewer (one of AK, MKE, MC). Disagreements regarding inclusion of full texts and data extraction were resolved through mutual discussion.

### Quality assessment

We assessed study quality using the National Institute for Health and Care Excellence (NICE) quality appraisal checklist.^16^ Studies were scored independently by two reviewers (two of AK, MGo, MKE, MC) with scoring disagreements resolved through mutual discussion. Internal and external validity were rated as poor, acceptable and very good based on NICE quality appraisal checklist results. We considered overall study quality to be high quality when both internal and external validity scores were rated as very good, adequate quality when either score was rated as acceptable and poor quality when either internal or external validity was rated as poor.

### Synthesis of study results

We grouped studies into three outcome areas: neurodevelopmental, cognition/academic achievement and mental health/behavioural outcomes (with some studies reporting outcomes from more than one category). We differentiated tools which measured general neurodevelopmental outcomes (and included a component of cognition but also include motor and speech domains) from those which specifically measured only cognition or academic language tests. We undertook a narrative synthesis of results within each of these areas as diverse outcomes and measurement tools precluded meta-analysis. We followed the Synthesis without meta-analysis (SWiM) reporting guidelines for analysing and reporting results.^17^

For neurodevelopmental studies we grouped studies and compared results by neurodevelopmental assessment tool used. For studies investigating cognition/academic achievement, we grouped those that measured IQ or executive function and those that measured either school or language performance/assessment results. For mental health/behaviour studies, we grouped studies that used behavioural assessment tools and then into groups by mental health condition or domain assessed.

For each study outcome where available, we recorded the unadjusted and adjusted results in cases and controls, reported effect sizes, and results from any statistical significance tests. When synthesising results by the groupings described above, we used vote counting by direction of effect to assess the number of studies which recorded differences between cases and controls. We summarised results in tables showing the number of studies with an effect on each outcome area. We also recorded the age group (childhood 0–10 years, adolescence 11–17 years, adulthood 18+ years) at which outcomes were measured to compare results between studies. Using SWiM guidelines we used vote counting in conjunction with study quality scores (which accounted for sample size, use of validated outcome tools/assessments and whether results were adjusted for confounders) to summarise the strength of conclusions.^17^ Where several high-quality studies in one grouped outcome area reported results of an association with malnutrition, we graded conclusions as strong evidence. Where several adequate and high-quality studies reported an association with malnutrition, but there were uncertainties or limitations of studies identified, we graded conclusions as moderate evidence. Where there were few studies identified or studies with mixed results or poor study quality, we graded associations as inconclusive.

## Results

### Study characteristics

**Figure 1:**
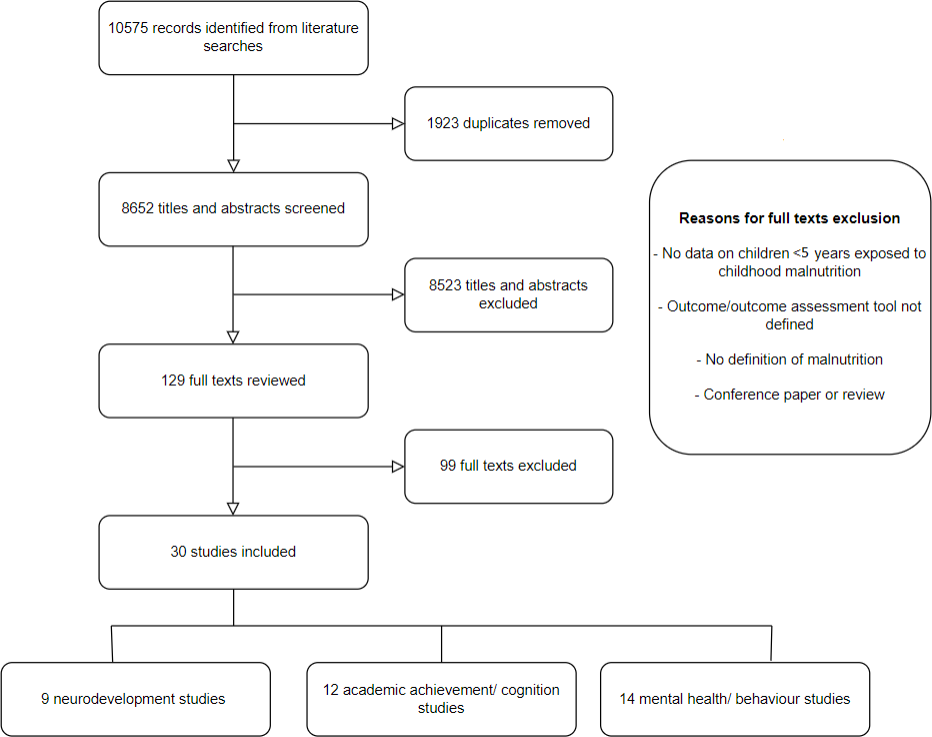
Flow chart of literature search.

Thirty studies met our selection criteria (figure 1).^18–47^ Full study characteristics are included in table 1. Studies published since 1995 were conducted across several countries in Africa, Asia and South America and included long-term cohorts recruited from 1967 onwards following participants through to adolescence and adulthood. Nine studies were part of the same Barbados Nutrition Study (BNS), a prospective lifelong cohort study assessing multiple outcomes at different follow-up points for children who suffered from malnutrition in the first year of life.^39–47^ Nine studies assessed neurodevelopmental outcomes, 12 studies assessed cognition or academic achievement (three from BNS) and 14 studies assessed mental health conditions or behaviour (eight from BNS). Study designs included cross-sectional, retrospective cohorts, prospective cohorts and randomised controlled trial studies. Neurodevelopmental studies were either cross-sectional or had a short follow-up time during childhood. The age at follow-up following exposure to malnutrition in other studies varied from childhood through to adulthood.

**Table 1:** Study characteristics

Study (year published)	Country (years conducted)	Study design (sample size)	Age exposed to malnutrition (age at follow-up if different)	Malnutrition definition (reference population)	Outcome	Outcome measurement tool
Chen *et al* (2021)^18^	China (2018)	Cross-sectional study (23 cases, 1270 controls)	3–5 years	w/h z score ≤2 (WHO)	Cognition/school achievement, Behaviour/mental health	WPPSI-IV SDQ
Mwene-Batu *et al* (2020)^19^	DRC (1988–2019)	Retrospective cohort (524 cases, 407 controls)	Median 41 months (median 22 years)	w/h z score ≤3 (WHO)	Cognition/school achievement, Behaviour/mental health	MMSE, Rosenberg Self-Esteem Scale, WHODAS
Asiki *et al* (2019)^20^	Uganda (1999–2011)	Retrospective cohort (12 cases with wasting, 145 cases recovered from wasting, 464 controls)	2–5 years (13–16 years)	w/h z score ≤2 (WHO)	Cognition/school achievement	Parental report of school years achieved
Dwivedi *et al* (2018)^21^	India (2014–15)	Cross-sectional study (102 cases, 101 controls)	6–30 months	WHO definition of severe acute malnutrition	Neurodevelopment	DASII (Indian modification of BSID)
Kang *et al* (2018)^22^	Bangladesh (2014), Bhutan (2011), Nepal (2015), Punjab, Pakistan (2016), Sindh, Pakistan (2015)	Cross-sectional study (31 037 participants)	36–59 months	w/h z score ≤2 (WHO)	Neurodevelopment	MICS ECDI
Lelijiveld *et al* (2018)^23^	Malawi (2006–2013)	Prospective cohort (171 cases, 155 controls)	Median 21.5 months(IQR 15–32) (9 years)	w/h<70% of the median (NCHS)	Cognition/school achievement	CANTAB, Parental report of school grade
Abessa *et al* (2017)^24^	Ethiopia (2011–13)	Cross-sectional study (310 cases, 310 controls)	Cases—mean 30.7 months (SD 15.2) controls—mean 29.6 months (SD 15.4)	w/h<70% of the median (NCHS), MUAC <110 mm, or bilateral pitting oedema due to malnutrition	Neurodevelopment, behaviour/mental Health	Denver II, ASQ
Sudfeld *et al* (2015)^25^	Tanzania (2010–14)	Randomised controlled trial (47 cases, 989 controls)	18–36 months	w/h z score ≤2 (WHO)	Neurodevelopment	BSID-III
De Grandis *et al* (2014)^26^	Argentina (not stated)	Retrospective cohort (25 cases, 28 controls)	0–2 years (5–12 years)	w/h z score ≤3 (or <70% expected) (WHO)	Behaviour/mental Health	Paediatric quality of life inventory
Malhi *et al* (2013)^27^	India (not stated)	Cross-sectional study (20 cases, 44 controls)	0–6 years	w/a z-score ≤2 (WHO)	Neurodevelopment	Indian development inventory
Bogale *et al* (2013)^28^	Ethiopia (2007)	Cross-sectional study (12 cases, 88 controls)	61.0 months ±3.0	w/a z-score ≤2 (WHO)	Cognition/school achievement	Raven’s CPM, KABC-II
Warsito *et al* (2012)^29^	Indonesia (not stated)	Cross-sectional study (5 cases, 48 controls)	3–5 years old	w/h z-score ≤2 (WHO)	Cognition/school achievement	Indonesian Department of National Education-Child Development Instrument
Nassar *et al* (2012)^30^	Egypt (not stated)	Cross-sectional study (33 cases, 30 controls)	3–6 years old	Wellcome classification (WHO)	Cognition/school achievement	Stanford-Binet-Intelligence-Scale (Arabic-translation) Receptive/Expressive/-Total-Language (Arabic-Language-Test)
Baker-Henningham *et al* (2009)^31^	Bangladesh (not stated)	Cross-sectional study (212 cases, 108 controls)	6–24 months old	w/a z-score ≤2 (NCHS)	Behaviour/mental Health	Temperament Questionnaire derived from existing validated instruments (modified by Wachs, Purdue University)
El-Khayat *et al* (2007)^32^	Egypt (2006)	Cross-sectional study (42 cases, 15 controls)	6–25 months	w/h z-score ≤2 (WHO)	Neurodevelopment	BSID-II
Liu *et al* (2004)^33^	Mauritius (1972-)	Prospective Cohort (235 cases, 807 controls)	3 years (8,11, 17 years)	Malnutrition and one of angular stomatitis, kwashiorkor, sparse thin hair, haemoglobin <8.5 g/dL	Behaviour/mental Health	Child Behaviour Questionnaire at age 8, Child Behaviour Checklist at age 11, Revised Behaviour Checklist at Age 17
Liu *et al* (2003)^34^	Mauritius (1972-)	Prospective Cohort (253 cases, 837 controls	3 years (11 years)	Malnutrition and one of angular stomatitis, kwashiorkor, sparse thin hair, haemoglobin <8.5g/dL	Cognition/school achievement	Bohem Test WISC Academic Tests Holborn-Reading Scale Trail Making Test
Drewett *et al* (2001)^35^	Ethiopia (not stated)	Prospective Cohort (97 cases, 100 controls)	2 years	w/a z-score <−1.88 (NCHS)	Neurodevelopment	BSID
Vazir *et al* (1998)^36^	India (not stated)	Cross-sectional study (1456 cases, 2212 controls)	0–6 years	<75% expected w/a (NCHS)	Neurodevelopment	ICMR Psychosocial Developmental Screening Test
Perales *et al* (1996)^37^	Chile (1987 – not stated)	Retrospective Cohort (40 cases, 40 controls)	0–2 years (8–10 years)	Protein energy malnutrition (defined by Sempe (1979) *et al*)	Cognition/school achievement	Continuous Performance Task Anstey Domino Test
Kaul *et al* (1995)^38^	India (not stated)	Cross-sectional study (50 cases, 102 controls)	0–12 months	Gomez classification—moderate malnutrition (NCHS)	Neurodevelopment	BSID
Barbados Nutrition Study						
Hock et al* (2018)^39^	Barbados (1977-not stated)	Prospective cohort (77 cases, 62 controls)	0–1 years (40–45 years)	Gomez classification	Behaviour/mental health	SCID-II-PQ, NEO PI-R FFM
Waber (1) et al* (2014)^40^	Barbados (1977–2010)	Prospective cohort (77 cases, 59 controls)	0–1 years (38 years)	Gomez classification	Cognition/school achievement	WASI, WRAT-III
Waber (2) et al* (2014)^41^	Barbados (1977–2010)	Prospective cohort (77 cases, 59 controls)	0–1 years (38 years)	Gomez classification	Cognition/school achievement, behaviour/mental Health	WAIS-III, D-KEFS, WRAML-II, Wisconsin card sorting, Metacognitive index, Behavioural regulation index
Galler et al* (2013)^42^	Barbados (2006–2010)	Prospective cohort (77 cases, 57 controls)	0–1 years (37–43 years)	Gomez classification	Behaviour/mental health	NEO-PI-R personality inventory
Galler (1) et al* (2012)^43^	Barbados (1967–2010)	Prospective cohort (80 cases, 65 controls)	0–1 years (40 years)	Gomez classification	Behaviour/mental Health	CAARS, CPT
Galler (2) et al* (2012)^44^	Barbados (1967–1985)	Prospective cohort (56 cases, 60 controls)	0–1 years (15 years old)	Gomez classification	Behaviour/mental Health	Child-Behaviour-Questionnaire, Teacher Behaviour Questionnaire
Waber et al* (2011)^45^	Barbados (1967–1985)	Prospective cohort (57 cases, 60 controls)	0–1 years (11–18 years)	Gomez classification	Cognition/school achievement and behaviour/mental health	WISC, Common entrance examination (local school test), Teacher-Behaviour Questionnaire, School functioning scale Minnesota-General-Adjustment-and Morale-Scale
Galler et al* (2011)^46^	Barbados (1967–1985)	Prospective cohort (109 cases, 107 controls)	0–1 years (9–17 years)	Gomez classification	Behaviour/mental health	Barbados-Child-Behaviour-Scale
Galler et al* (2010)^47^	Barbados (1967–1985)	Prospective cohort (116 cases, 61 controls)	0–1 years (11–17 years old)	Gomez classification	Behaviour/mental health	Minnesota-General-Adjustment-and Morale-Scale

*Studies are drawn from the same Barbados Nutrition Study Cohort.

ASQ, Ages and Stages Questionnaire; BSID, Bayley scales of infant development; CAARS, Attention-domain: Conners-ADHD-Rating-Scales; CANTAB, Cambridge Neuropsychological Test Automated Battery; CPT, Conners-Continuous-Performance Test; DASII, Developmental Assessment Scale of Indian Infants; D-KEFS, Delis-Kaplan Executive Function System; DRC, Democratic Republic of Congo; ICMR, Indian Council Medical Research Psychosocial Developmental Screening Test; KABC-II, Kaufman Assessment Battery for Children-II; MDAT, Malawi Developmental Assessment Tool; MICS ECDI, Multiple Indicator Cluster Survey Early Child Development Index; MMSE, Mini Mental State Exam; MUAC, Mid Upper Arm Circumference; NCHS, National Centre for Health Statistics reference population; NEO PI-R FFM, NEO Personality Inventory-Revised derived Five-Factor Model; Raven’s CPM, Raven’s Coloured Progressive Matrices; SCID-II-PQ, Structured Clinical Interview for DSM-IV Axis II Personality Disorders Personality Questionnaire; SDQ, Strengths and Difficulties Questionnaire; WAIS-III, Wechsler Adult Intelligence Scale-III; WASI, Adult IQ: Weschler Abbreviated Scale of Intelligence – Vocabulary and Matrix Reasoning subsets; w/h, Weight-for-Height; WHO, WHO reference population; WHODAS, WHO Disability Assessment Schedule; WISC, Weschler Intelligence Scale; WPPSI-IV, Wechsler Preschool and Primary Scale of Intelligence Fourth Edition; WRAML-II, Wide Range Assessment of Memory and Learning-II; WRAT-III, Academic achievement: Wide Range Achievement Test-III-Reading Spelling and Calculation subsets.

Study quality scoring is included in online supplemental table 1. Twenty-three studies had very good external validity, with the seven others apart from one with acceptable external validity. This was due to most providing good descriptions of study settings, populations and selection criteria. Fourteen studies had very good internal validity, 12 had acceptable internal validity and four studies had poor internal validity. Studies often scored poorly where important confounding variables such as socioeconomic status were not accounted for in analyses, sample size was small or study designs were poor or poorly described, leading to potential biases. Overall, 14 studies were rated as high quality, 12 were adequate quality and four were poor quality.

### Malnutrition and neurodevelopment

We identified nine studies assessing neurodevelopmental outcomes of malnutrition (table 2). A summary of results from these studies is included in online supplemental table 2. Five studies used the Bayley Scales of Infant Development (BSID). Three of these were adequate-quality studies which found impaired neurodevelopment in those with malnutrition unadjusted for confounding variables such as socioeconomic status and family characteristics.^21 32 38^ Two high-quality studies using BSID found impaired neurodevelopment in those with malnutrition but one found that differences were no longer significant when adjusting for differences in current weight during follow-up at 2 years old.^25 35^ These differences in BSID scores where present across studies for both mental and psychomotor subscales. A further high-quality study using the Denver-II tool found children with malnutrition had lower scores across fine motor, gross motor, language and personal-social domains compared with controls.^24^ One adequate-quality study using a neurodevelopment tool developed in India (Indian Council Medical Research Psychosocial Developmental Screening Test) found poorer development outcomes for those with malnutrition.^36^ Another adequate-quality study using UNICEF Multiple Indicator Cluster Surveys (MICS) Early Childhood Development Indicators (ECDI) did not find an effect of malnutrition on learning-cognition or socioemotional development.^22^ This was carried out on a large population sample from five country surveys in Asia, but the MICS ECDI tool consists of few basic developmental items which span a large age range. This is notably different to the detailed neurodevelopmental assessments by trained assessors used in most other studies. A poor-quality study using an Indian specific development tool (Indian Development Inventory), found poorer neurodevelopment scores across social, adaptive, motor, communication and cognitive subscales in cases, but this was no longer significant when adjusted for confounding variables. The results from high-quality and adequate-quality studies provides strong evidence of an association between malnutrition in childhood and impaired neurodevelopment across multiple developmental domains.

**Table 2:** Summary of results from studies assessing the effect of malnutrition on neurodevelopment

Study (quality) country (outcome age)	Neurodevelopmental assessment tool				
	BSID	MICS ECDI	Denver	IDI	ICMR
Dwivedi *et al* 2018 (+) India (childhood)^21^	Effect (unadjusted)*				
Kang *et al* 2018 (+) multiple in Asia (childhood)^22^		No effect (adjusted)			
Abessa *et al* 2017 (++) Ethiopia (childhood)^24^			Effect (adjusted)		
Sudfeld *et al* 2015 (++) Tanzania (childhood)^25^	Effect (adjusted)				
Malhi *et al* 2013 (-) India (childhood)^27^				No effect (adjusted)	
El-Khayat *et al* 2007 (+) Egypt (childhood)^32^	Effect (unadjusted)				
Drewett *et al* 2001 (++) Ethiopia (childhood)^35^	No effect (adjusted)				
Vazir *et al* 1998 (+) India (childhood)^36^					Effect (unadjusted)
Kaul et al 1995 (+) India (childhood)^38^	Effect (unadjusted)				

Effect (marked in green) —statistically significant difference in neurodevelopment between cases and controls.

No effect (marked in red)—no difference, or statistically insignificant difference, or statistically insignificant difference after adjusting for confounding variables, in neurodevelopment between cases and controls.

Adjusted—results adjusted for confounding variables.

Unadjusted—results unadjusted for confounding variables.

++=high quality.

+=adequate quality.

-=poor quality.

*Indian modification of BSID.

BSID, Bayley Scales of Infant Development; ICMR, Indian Council Medical Research Psychosocial Developmental Screening Test; IDI, Indian Developmental Inventory; MICS ECDI, Multiple Indicator Cluster Survey Early Child Development Index.

### Malnutrition and cognition/academic achievement

We identified 12 studies (three from BNS) assessing cognitive outcomes of malnutrition (table 3). A summary of results from these studies is included in online supplemental table 3. Seven studies assessed academic performance, with three based on self-report of school performance or school year achieved by age at follow-up (a measure in one Malawian study where school year progression is based on performance^23^), and four based on tests of school skill such as mathematics or country specific language tests (online supplemental table 3).^19 20 23 30 34 40 45^ Three studies were high quality, three were adequate quality and one was poor quality. All studies (two from BNS) found worse school/academic performance in those exposed to malnutrition in childhood compared with controls. The results from high-quality and adequate-quality studies identified provides strong evidence of an association between exposure to malnutrition and impaired academic performance/achievement in childhood and adolescence.

**Table 3:** Summary of results from studies assessing the effect of malnutrition on cognition and academic achievement

Study (quality) (outcome age) Country	School, academic or language performance	Executive function/intelligence (assessment tool)
Chen *et al* 2021 (++) (childhood) China^18^		No effect (adjusted) (WPPSI-IV)
Mwene-Batu *et al* 2020 (+) (adulthood) DRC^19^	Effect (unadjusted)	Effect (unadjusted) (MMSE)
Asiki et al 2018 (++) (adolescence) Uganda**20**	Effect (adjusted)	
Lelijiveld *et al* 2018 (++) (childhood) Malawi^23^	Effect (adjusted)	No effect (adjusted) (CANTAB)
Bogale *et al* 2013 (++) (childhood) Ethiopia^28^		Effect (adjusted) (Raven’s CPM, KABC-II)
Warsito *et al* 2012 (-) (childhood) Indonesia^29^		No effect (unadjusted) (Indonesian Department of National Education – child development instrument score)
Nassar *et al* 2012 (-) (childhood) Egypt^30^	Effect (unadjusted)	Effect (unadjusted) (Stanford-Binet-Intelligence-Scale)
Liu *et al* 2003 (++) (adolescence) Mauritius^34^	Effect (adjusted)	Effect (adjusted) (Bohem Test, Trail Making Test, WISC)
Perales *et al* 1996 (+) (childhood) Chile^37^		Effect (unadjusted) [Continuous Performance Task, Anstey Domino test]
Barbados Nutrition Study		
Waber (1) *et al* 2014* (++) (adulthood) Barbados**40**	Effect (adjusted)	Effect (adjusted) (WASI)
Waber (2) *et al* 2014* (++) (adulthood) Barbados**41**		Effect (adjusted) [WAIS-III, D-KEFS, WRAML-2, Wisconsin card sorting, Metacognitive index]
Waber *et al* 2011* (+) (adolescence) Barbados^45^	Effect (adjusted)	Effect (adjusted) (WISC)

Effect (marked in green) —statistically significant difference in academic achievement/cognition between cases and controls.

No effect (marked in red)—no difference, or statistically insignificant difference, or statistically insignificant difference after adjusting for confounding variables, in academic achievement/cognition between cases and controls.

Adjusted—results adjusted for confounding variables.

Unadjusted—results unadjusted for confounding variables.

++=high quality.

+=adequate quality.

-=poor quality.

*Studies are drawn from the same Barbados Nutrition Study.

CANTAB, Cambridge Neuropsychological Test Automated Battery; D-KEFS, Delis-Kaplan Executive Function System; KABC-II, Kaufman Assessment Battery for Children-II; MMSE, Mini-Mental State Exam; Raven’s CPM, Raven’s Coloured Progressive Matrices; WAIS-III, Wechsler Adult Intelligence Scale-III; WASI, Adult IQ: Wechsler Abbreviated Scale of Intelligence-Vocabulary and Matrix Reasoning subsets; WISC, Weschler Intelligence Scale for Children; WPPSI-IV, Wechsler Preschool and Primary Scale of Intelligence Fourth Edition; WRAML-II, Wide Range Assessment of Memory and Learning-II.

Eleven studies investigated cognition using several tools assessing intelligence and executive function (table 3, online supplemental table 3). Eight of these studies (three from BNS) found impaired intelligence/executive function in those exposed to malnutrition in childhood compared with controls.^19 28 30 34 37 40 41 45^ Two high-quality studies found no significant association between malnutrition and cognition.^18 23^ One study from China found no difference between cases and controls using Wechsler Pre-school and Primary Scale of Intelligence Fourth Edition, and the other from Malawi found the differences seen between cases and controls using CANTAB (the Cambridge Neuropsychological Test Automated Battery) were no longer significant when adjusting for confounding variables including HIV, stunting, socioeconomic status and household characteristics. Of the studies which found a significant difference in cognition between cases and controls, there were four high-quality studies (two from BNS).^28 34 40 41^ These four high-quality studies all used different cognitive assessment tools, including Kauffman-ABC, Bohem Test, Trail Making Test and Wechsler Scale of Intelligence (table 3), and found poorer scores in those exposed to malnutrition compared with controls. Three adequate-quality studies (one from BNS) using different cognitive assessment tools, including Mini-Mental State Exam, Wechsler Scale of Intelligence and Anstey Domino test (table 3) also found those exposed to childhood malnutrition had impaired cognition compared with controls, however, only one of these studies adjusted for any confounding variables (BNS study which adjusted for childhood standard of living).^19 37 45^ The results from high-quality and adequate-quality studies provide moderate evidence of an association between exposure to malnutrition in childhood and impaired cognition, but definitive conclusions are limited by mixed results from two high-quality studies showing no association after adjusting for important confounders such as HIV and socioeconomic status and results from several adequate-quality studies which failed to adjust for any confounding variables.

### Malnutrition and mental health/behaviour

We identified 14 studies (8 from BNS) assessing mental health and behavioural outcomes of malnutrition (table 4). A summary of results from these studies is included in online supplemental table 4. Seven studies (three from BNS) assessed behaviour using different behavioural rating tools including the Strengths and Difficulties Questionnaire (SDQ), Child Behaviour Checklist, Ages and Stages (socioemotional questions) and other adapted behavioural questionnaires (table 4).^18 24 31 33 41 44 46^ Five of these studies, four of which were rated as high quality, found significantly higher behavioural problems in those exposed to malnutrition in childhood.^24 31 33 41 46^ One high-quality study found no difference in behavioural scores between cases and controls using SDQ and one adequate-quality study looking at conduct problems using the child behaviour questionnaire found differences in behaviour were no longer significant when adjusting for confounding variables including childhood standard of living. The results from adequate-quality and high-quality studies provide moderate evidence of an association between exposure to malnutrition in childhood and increased behavioural problems but is limited by some mixed results in high-quality studies and potential confounding in some studies reporting an association.

**Table 4:** Summary of results from studies assessing the effect of malnutrition on mental health and behaviour

Study (quality) (outcome age) Country	Behavioural problems (tool)	Self-esteem (tool)	Social-related disability (tool)	Quality of life (tool)	Personality (tool)	Attention (tool)	Morale (tool)
Chen *et al* 2021 (++) (childhood) China^18^	No effect (adjusted) (SDQ)						
Mwene-Batu *et al* 2020 (+) (adulthood) DRC^19^		Effect (unadjusted) (Rosenberg Self-Esteem Scale)	No effect (unadjusted) (WHODAS)				
Abessa *et al* 2017 (++) (childhood) Ethiopia^24^	Effect (adjusted) (ASQ:SE)						
De Grandis *et al* 2014 (-) (childhood) Argentina^26^				Effect (unadjusted) (Paediatric quality of life inventory)			
Baker-Henningham *et al* 2009 (++) (childhood) Bangladesh^31^	Effect (adjusted) (Wachs temperament questionnaire)						
Liu *et al* 2004 (+) (childhood, adolescence) Mauritius^33^	Effect (adjusted) (Child Behaviour Checklist)						
Barbados Nutrition Study							
Hock *et al* 2018* (+) (adulthood) Barbados^39^					Mixed (adjusted) [SCID-II-PQ, NEO PI-R FFM]		
Waber (2) *et al* 2014* (++) (adulthood) Barbados**41**	Effect (adjusted) (Behavioural regulation index)						
Galler *et al* 2013* (+) (adulthood) Barbados^42^					Effect (adjusted) (NEO-PI-R)		
Galler (1) *et al* 2012* (+) (adulthood) Barbados**43**						Mixed (adjusted) (CAARS, CPT)	
Galler (2) *et al* 2012* (+) (adolescence) Barbados**44**	No effect (adjusted) (Conduct: CBQ, Teacher behaviour questionnaire)						
Waber *et al* 2011* (+) (adolescence) Barbados^45^						No effect (adjusted) (Attention: CBQ)	No effect (adjusted) (Minnesota Adjustment and Morale Scale)
Galler *et al* 2011* (++) (adolescence) Barbados^46^	Effect (adjusted) (BCBS)						
Galler *et al* 2010* (++) (adolescence) Barbados^47^							Effect (adjusted) (Minnesota Adjustment and Morale Scale)

Effect (marked in green)—statistically significant difference in mental health/behaviour between cases and controls.

No effect (marked in red)—no difference, or statistically insignificant difference, or statistically insignificant difference after adjusting for confounding variables, in mental health/behaviour between cases and controls.

Mixed (marked in blue)-mixture of results in the same paper showing statistically significant differences of certain outcome measurements but not other

Adjusted—results adjusted for confounding variables.

Unadjusted—results unadjusted for confounding variables.

++=high quality.

+=adequate quality.

-=poor quality.

*Studies are drawn from the same Barbados Nutrition Study.

ASQ-SE, Ages and Stages Questionnaire Socio Emotional; Attention-domain, Conners-ADHD-Rating-Scales; BCBS, Barbados Child Behaviour Scale; CAARS, Attention-domain: Conners-ADHD-Rating-Scales; CBQ, Child Behaviour Questionnaire; CPT, Conners-Continuous-Performance Test; NEO PI-R, NEO Personality Inventory-Revised; NEO PI-R FFM, NEO Personality Inventory-Revised derived Five-Factor Model; SCID-II-PQ, Structured Clinical Interview for DSM-IV Axis II Personality Disorders Personality Questionnaire; SDQ, Strengths and Difficulties Questionnaire; WHODAS, WHO Disability Assessment Schedule.

Seven studies (five from BNS) investigated different mental health outcomes (table 4).^19 26 39 42 43 45 47^ Studies indicated possible associations with self-esteem, quality of life, personality disorders, attention deficits and low morale. There were small numbers of studies investigating specific mental health domains and there were significant study limitations in several studies such as failure to adjust for confounders and the use of multiple tools and domains within individual studies, increasing the risk of type 1 error. The results from the studies identified provide inconclusive evidence regarding possible associations between exposure to malnutrition in childhood and poorer mental health outcomes.

## Discussion

Our review finds strong evidence that exposure to malnutrition in childhood impairs neurodevelopment and academic achievement. There is moderate evidence that childhood malnutrition is associated with impaired cognition and is associated with more behavioural problems throughout childhood and adolescence. However, there is uncertainty around the relative contributions of malnutrition and other associated factors (such as childhood adversity, HIV-exposure, socioeconomic status and household characteristics) to these outcomes. Research investigating mental health outcomes in children with malnutrition is inconclusive and there are few studies investigating specific mental health domains such as depression. These results have implications for policy-makers surrounding the long-term care needs of those treated for childhood malnutrition and there is a need for research exploring how nutritional therapies and social interventions affect these outcomes.

Due to study heterogeneity, we were unable to perform a meta-analysis of any results. We therefore, used published guidelines to synthesise results and determine the strength of evidence for each outcome area. Despite this, there are still limitations given potential publication bias of positive associations between malnutrition and the outcomes investigated. Our review, however, builds on previously published evidence which has suggested links between malnutrition and poorer IQ levels, cognitive function, school achievement and greater behavioural problems.^11^ Our findings strengthen the evidence base regarding these associations as previous findings were limited by difficulties in interpreting retrospective case control studies, but our review includes evidence from prospective studies which have sought to minimise these biases. There are however several poor-quality or adequate-quality studies we identified which failed to account for important confounders such as socioeconomic status and family characteristics. Of the 30 studies we identified, 9 were from the same prospective cohort study in Barbados, with many of these publications from the same cohort testing multiple cognitive, behavioural and mental health outcomes. This may have increased the likelihood of type 1 error. For example, different studies from the same cohort found mixed results regarding morale and personality disorder scores when different assessment tools were used (table 4).^43 45 47^ However, we accounted for this when assessing the strength of conclusions. We focused on studies from low-income and middle-income countries where there is a high disease burden from severe malnutrition. Results are therefore applicable to similar settings and outcomes may differ in children with malnutrition in high-middle-income settings where other societal factors can alter development trajectories. Differentiating the relative effects of malnutrition and subsequent socioeconomic factors is therefore difficult from studies in this review but impaired cognition due to malnutrition in high-income settings has been found in other studies such as those looking at outcomes from the ‘Dutch winter hunger’.^48^

Impaired neurodevelopment in childhood is likely linked to subsequently poorer academic achievement in childhood and adolescence and may also be linked to increased behavioural problems seen in some studies.^49^ The studies assessing IQ/executive function outcomes spanned childhood through to adulthood and there was moderate evidence of a link between malnutrition and cognition. Two high-quality studies showed no effect^18 23^ and other studies which showed an effect did not adjust for any confounding variables.^19 30 37^ There are therefore remaining questions over how much an early insult to neurodevelopment from malnutrition affects future cognition and functioning, and to what extent other related environmental factors such as prenatal nutrition, family characteristics and infections contribute to these outcomes.^50 51^ Several studies adjusted for sex when analysing data but there are insufficient studies with stratified data in this review to comment confidently on possible sex-specific differences in outcomes. A recent systematic review found that sex can significantly influence outcomes with higher odds of boys being wasted, underweight and stunted than girls.^52^ The review also found geographical variation in outcomes and the reasons for sex-specific differences remain unclear with both plausible biological and social causes. This is therefore an important area of future research with regards to the outcomes we investigate in this study.^53^ Mental health outcomes such as depression and inattention are likely to be influenced by similar confounding variables, and whether early insults from malnutrition independently contribute to poor mental health outcomes in later life is yet to be established. There is also uncertainty around the effect of nutritional therapy on long-term cognition and functioning and there has been significant interest in catch-up growth during key periods.^54^ For example, previous research has indicated that weight gain in the first 2 years of life predicts schooling outcomes and there is ongoing work to determine the optimum speed and regimen of nutritional therapy to maximise long-term outcomes.^55 56^ However, even if some of the effects we report are due to confounding, evidence is clear from our review that children who experience an episode of malnutrition in childhood are at high risk of poorer development, behaviour and cognition. Specific adversities prevalent in low-income and middle-income settings related to low socioeconomic status are areas of potential intervention which may improve outcomes. These can target areas such as parenting, schooling, poverty alleviation, ending child exploitation and labour, all of which are likely to influence outcomes. Policy-makers should therefore prioritise targeted support both nutritionally and societally for these vulnerable children to optimise life chances after recovery.

While there is still uncertainty around the relative contributions of associated medical and social factors, there is increasing evidence from this review that the early impacts of malnutrition are related to worse academic, cognitive and behavioural outcomes compared with well-nourished peers. Preventing and decreasing childhood malnutrition is therefore of key importance to prevent serious long-term neurocognitive issues in affected children, particularly given the ongoing high malnutrition prevalence in many low-income and middle-income settings. Further research is needed on how to optimise treatment and to best support ongoing care for survivors to improve outcomes in the long term.

## Data Availability

All data relevant to the study are included in the article or uploaded as online supplemental information.
