# Supplementary material for: Neurodevelopmental, cognitive, behavioural and mental health impairments following childhood malnutrition: a systematic review
Source: BMJ Glob Health. 2022 Jul 6;7(7):e009330. doi: 10.1136/bmjgh-2022-009330 (PMC9260807; doi:10.1136/bmjgh-2022-009330)
Supplement: Supplementary data [file bmjgh-2022-009330supp001.pdf]

Supplementary Appendix 1 – Search strategies

EMBASE

1. severe acute malnutrition.ti,ab.
2. acute severe malnutrition.ti,ab.
3. acute malnutrition.ti,ab.
4. (acute adj2 malnutrition).ti,ab.
5. "sever* acute* maln*".ti,ab.
6. protein-energy malnutrition.ti,ab.
7. protein calorie malnutrition.ti,ab.
8. (protein malnutrition or energy malnutrition or energy-protein malnutrition).ti,ab.
9. "acute* maln*".ti,ab.
10. "kwas?io?kor*".ti,ab.
11. "marasm*".ti,ab.
12. "marasm* kwas?io?kor*".ti,ab.
13. wasting.ti,ab.
14. "acute wast*".ti,ab.
15. wasted.ti,ab.
16. (acute adj2 wasting).ti,ab.
17. (severe adj2 wasting).ti,ab.
18. acute undernutrition.ti,ab.
19. (acute adj2 undernutrition).ti,ab.
20. severe undernutrition.ti,ab.
21. (severe adj2 undernutrition).ti,ab.
22. "mid* upper arm circumference".ti,ab.
23. (mid* upper arm circumference adj "115").ti,ab.
24. (mid* upper arm circumference adj "110").ti,ab.
25. MUAC.ti,ab.
26. "acute* undernourish*".ti,ab.
27. "acut* emaciat*".ti,ab.
28. "acut* maln*".ti,ab.
29. *malnutrition/
30. exp kwashiorkor/
31. exp marasmus/
32. exp wasting syndrome/
33. exp protein calorie malnutrition/
34. *nutritional disorder/
35. 1 or 2 or 3 or 4 or 5 or 6 or 7 or 8 or 9 or 10 or 11 or 12 or 13 or 14 or 15 or 16 or 17 or 18 or 19 or 20 or 21 or 22 or 23 or 24 or 25 or 26 or 27 or 28 or 29 or 30 or 31 or 32 or 33 or 34
36. infant/
37. child/

38. exp toddler/
39. "child*".ti,ab.
40. "infan*".ti,ab.
41. "pre-school*".ti,ab.
42. "preschool*".ti,ab.
43. "toddler*".ti,ab.
44. 36 or 37 or 38 or 39 or 40 or 41 or 42 or 43
45. 35 and 44
46. "child* developm* ".ti,ab.
47. "child* neurodevelopm*".ti,ab.
48. "neurodevelopm*".ti,ab.
49. (socio-emotion* develop* or socioemotion* develop*).ti,ab.
50. "social developm*".ti,ab.
51. "emotion* developm*".ti,ab.
52. (sensorymotor developm* or sensory-motor developm*).ti,ab.
53. "motor developm*".ti,ab.
54. "motor neurodevelopm*".ti,ab.
55. exp child development/ or exp postnatal development/
56. 46 or 47 or 48 or 49 or 50 or 51 or 52 or 53 or 54 or 55
57. exp cognition assessment/ or exp cognition/
58. exp learning/ or exp learning disorder/
59. exp intelligence quotient/ or exp intelligence/
60. exp language development/ or exp human development/ or exp mental development/
61. *brain function/ or *central nervous system function/
62. cognition.ti,ab.
63. "cogniti* performance".ti,ab.
64. intelligence.ti,ab.
65. IQ.ti,ab.
66. "executive function*".ti,ab.
67. reasoning.ti,ab.
68. language.ti,ab.
69. attention.ti,ab.
70. memory.ti,ab.
71. memory.ti,ab.
72. learning.ti,ab.
73. early learning.ti,ab.
74. information processing.ti,ab.
75. literacy.ti,ab.
76. reading.ti,ab.
77. math.ti,ab.

78. 57 or 58 or 59 or 60 or 61 or 62 or 63 or 64 or 65 or 66 or 67 or 69 or 70 or 71 or 72 or 73 or 74 or 75 or 76 or 77
79. education.ti,ab.
80. "school* achievement".ti,ab.
81. "school* performance".ti,ab.
82. school retention.ti,ab.
83. academic.ti,ab.
84. pre-academic.ti,ab.
85. schooling.ti,ab.
86. exp academic achievement/
87. 79 or 80 or 81 or 82 or 83 or 84 or 85 or 86
88. exp behavior/
89. exp mental health/
90. exp psychological aspect/ or exp neuropsychology/ or exp attention/ or exp cognitive defect/ or exp mental disease/
91. exp depression/
92. *hyperactivity/ or *psychomotor disorder/
93. exp attention deficit disorder/
94. "behavi?r* problem*".ti,ab.
95. "emotion* problem*".ti,ab.
96. "temperament*".ti,ab.
97. self regulation.ti,ab.
98. attachment.ti,ab.
99. self esteem.ti,ab.
100. self efficacy.ti,ab.
101. "social competen*".ti,ab.
102. "peer relationship*".ti,ab.
103. pro-social behavi?r.ti,ab.
104. hyperactivity.ti,ab.
105. impulsivity.ti,ab.
106. attention* deficit hyperactivity disorder*.ti,ab.
107. agression.ti,ab.
108. 88 or 89 or 90 or 91 or 92 or 93 or 94 or 95 or 96 or 97 or 98 or 99 or 100 or 101 or 102 or 103 or 104 or 105 or 106 or 107
109. 56 or 78 or 90 or 108
110. 45 and 109
111. limit 110 to (human and yr="1995 -Current")

MEDLINE

1. "acute severe malnutrition".ti,ab.

Kirolos A, *et al. BMJ Global Health* 2022; 7:e009330. doi: 10.1136/bmjgh-2022-009330

2. severe acute malnutrition.ti,ab.
3. acute malnutrition.ti,ab.
4. (acute adj2 malnutrition).ti,ab.
5. "sever* acute* maln*".ti,ab.
6. protein-energy malnutrition.ti,ab.
7. acute severe malnutrition.ti,ab.
8. (protein malnutrition or energy malnutrition or energy-protein malnutrition).ti,ab.
9. "acute* maln*".ti,ab.
10. "kwas?io?kor*".ti,ab.
11. "marasm*".ti,ab.
12. "marasm* kwas?io?kor*".ti,ab.
13. wasting.ti,ab.
14. "acute wast*".ti,ab.
15. wasted.ti,ab.
16. (acute adj2 wasting).ti,ab.
17. (severe adj2 wasting).ti,ab.
18. acute undernutrition.ti,ab.
19. (acute adj2 undernutrition).ti,ab.
20. severe undernutrition.ti,ab.
21. (severe adj2 undernutrition).ti,ab.
22. "mid* upper arm circumference".ti,ab.
23. (mid* upper arm circumference adj "115").ti,ab.
24. (mid* upper arm circumference adj "110").ti,ab.
25. MUAC.ti,ab.
26. "acute* undernourish*".ti,ab.
27. "acut* maln*".ti,ab.
28. exp child nutrition disorders/ or exp deficiency diseases/ or exp starvation/ or *wasting syndrome/
29. exp Protein-Energy Malnutrition/
30. exp Infant Nutrition Disorders/
31. 1 or 2 or 3 or 4 or 5 or 6 or 7 or 8 or 9 or 10 or 11 or 12 or 13 or 14 or 15 or 16 or 17 or 18 or 19 or 20 or 21 or 22 or 23 or 24 or 25 or 26 or 27 or 28 or 29 or 30
32. "child*".ti,ab.
33. "infan*".ti,ab.
34. "pre-school*".ti,ab.
35. "preschool*".ti,ab.
36. "toddler*".ti,ab.
37. exp Infant/
38. exp Child/
39. Child, Preschool/
40. 32 or 33 or 34 or 35 or 36 or 37 or 38 or 39

41. 31 and 40
42. "child* developm* ".ti,ab.
43. "child* neurodevelopm*".ti,ab.
44. "neurodevelopm*".ti,ab.
45. (socio-emotion* develop* or socioemotion* develop*).ti,ab.
46. "social developm*".ti,ab.
47. "emotion* developm*".ti,ab.
48. (sensorymotor developm* or sensory-motor developm*).ti,ab.
49. "motor developm*".ti,ab.
50. "motor neurodevelopm*".ti,ab.
51. exp Child Development/
52. 42 or 43 or 44 or 45 or 46 or 47 or 48 or 49 or 50 or 51
53. cognition.ti,ab.
54. "cogniti* performance".ti,ab.
55. intelligence.ti,ab.
56. IQ.ti,ab.
57. "executive function*".ti,ab.
58. reasoning.ti,ab.
59. language.ti,ab.
60. attention.ti,ab.
61. memory.ti,ab.
62. learning.ti,ab.
63. early learning.ti,ab.
64. information processing.ti,ab.
65. literacy.ti,ab.
66. reading.ti,ab.
67. math.ti,ab.
68. *cognition/ or exp cognitive reserve/ or exp comprehension/ or exp executive function/ or exp learning/
69. exp Mild Cognitive Impairment/
70. *Intelligence/
71. *language development/ or *child language/
72. 53 or 54 or 55 or 56 or 57 or 58 or 59 or 60 or 61 or 62 or 63 or 64 or 65 or 66 or 67 or 68 or 69 or 70 or 71
73. education.ti,ab.
74. "school* achievement".ti,ab.
75. "school* performance".ti,ab.
76. school retention.ti,ab.
77. academic.ti,ab.
78. pre-academic.ti,ab.
79. schooling.ti,ab.

Kirolos A, *et al. BMJ Global Health* 2022; 7:e009330. doi: 10.1136/bmjgh-2022-009330

80. exp Educational Status/
81. 73 or 74 or 75 or 76 or 77 or 78 or 79 or 80
82. "behavi?r* problem*".ti,ab.
83. "emotion* problem*".ti,ab.
84. "temperament*".ti,ab.
85. self regulation.ti,ab.
86. attachment.ti,ab.
87. self esteem.ti,ab.
88. self efficacy.ti,ab.
89. "social competen*".ti,ab.
90. "peer relationship*".ti,ab.
91. pro-social behavi?r.ti,ab.
92. hyperactivity.ti,ab.
93. impulsivity.ti,ab.
94. attention* deficit hyperactivity disorder*.ti,ab.
95. aggression.ti,ab.
96. exp Child Behavior Disorders/
97. exp Mental Health/
98. *psychology/ or exp psychology, adolescent/ or exp psychology, child/
99. exp Depression/
100. exp Attention Deficit Disorder with Hyperactivity/
101. *Anxiety Disorders/
102. 82 or 83 or 84 or 85 or 86 or 87 or 88 or 89 or 90 or 91 or 92 or 93 or 94 or 95 or 96 or 97 or 98 or 99 or 100 or 101
103. 52 or 72 or 81 or 102
104. 41 and 103
105. limit 104 to (humans and yr="1995 -Current")

GLOBAL HEALTH

1. severe acute malnutrition.ti,ab.
2. acute severe malnutrition.ti,ab.
3. acute malnutrition.ti,ab.
4. (acute adj2 malnutrition).ti,ab.
5. "sever* acute* maln*".ti,ab.
6. protein-energy malnutrition.ti,ab.
7. protein calorie malnutrition.ti,ab.
8. (protein malnutrition or energy malnutrition or energy-protein malnutrition).ti,ab.
9. "acute* maln*".ti,ab.
10. "kwas?io?kor*".ti,ab.
11. "marasm*".ti,ab.

12. "marasm* kwas?io?kor*".ti,ab.
13. wasting.ti,ab.
14. "acute wast*".ti,ab.
15. wasted.ti,ab.
16. (acute adj2 wasting).ti,ab.
17. (severe adj2 wasting).ti,ab.
18. acute undernutrition.ti,ab.
19. (acute adj2 undernutrition).ti,ab.
20. severe undernutrition.ti,ab.
21. (severe adj2 undernutrition).ti,ab.
22. "mid* upper arm circumference".ti,ab.
23. (mid* upper arm circumference adj "115").ti,ab.
24. (mid* upper arm circumference adj "110").ti,ab.
25. MUAC.ti,ab.
26. "acute* undernourish*".ti,ab.
27. "acut* maln*".ti,ab.
28. emaciat*.ti,ab.
29. nutritional disorders/ or malnutrition/ or exp protein energy malnutrition/ or exp undernutrition/ or exp nutritional oedema/
30. wasting disease/ or exp emaciation/
31. 1 or 2 or 3 or 4 or 5 or 6 or 7 or 8 or 9 or 10 or 11 or 12 or 13 or 14 or 15 or 16 or 17 or 18 or 19 or 20 or 21 or 22 or 23 or 24 or 25 or 26 or 27 or 28 or 29 or 30
32. "child*".ti,ab.
33. "infan*".ti,ab.
34. "pre-school*".ti,ab.
35. "preschool*".ti,ab.
36. "toddler*".ti,ab.
37. exp infants/
38. exp preschool children/ or children/
39. 32 or 33 or 34 or 35 or 36 or 37 or 38
40. 31 and 39
41. "child* developm* ".ti,ab.
42. "child* neurodevelopm*".ti,ab.
43. "neurodevelopm*".ti,ab.
44. (socio-emotion* develop* or socioemotion* develop*).ti,ab.
45. "social developm*".ti,ab.
46. "emotion* developm*".ti,ab.
47. (sensorymotor developm* or sensory-motor developm*).ti,ab.
48. "motor developm*".ti,ab.
49. child development/ or exp early childhood development/ or exp infant development/ or exp psychomotor development/

50. 41 or 42 or 43 or 44 or 45 or 46 or 47 or 48 or 49
51. cognition.ti,ab.
52. "cogniti* performance".ti,ab.
53. intelligence.ti,ab.
54. IQ.ti,ab.
55. "executive function*".ti,ab.
56. reasoning.ti,ab.
57. language.ti,ab.
58. attention.ti,ab.
59. memory.ti,ab.
60. learning.ti,ab.
61. early learning.ti,ab.
62. information processing.ti,ab.
63. literacy.ti,ab.
64. reading.ti,ab.
65. math.ti,ab.
66. exp mental ability/ or exp cognitive development/ or exp memory/
67. exp learning/
68. 51 or 52 or 53 or 54 or 55 or 56 or 57 or 58 or 59 or 60 or 61 or 62 or 63 or 64 or 65 or 66 or 67
69. education.ti,ab.
70. "school* achievement".ti,ab.
71. "school* performance".ti,ab.
72. school retention.ti,ab.
73. academic.ti,ab.
74. schooling.ti,ab.
75. preacademic.ti,ab.
76. education/ or exp early childhood education/ or exp elementary education/ or exp extension education/ or exp higher education/ or exp primary education/ or exp professional education/ or exp secondary education/ or exp academic achievement/ or exp educational performance/
77. 69 or 70 or 71 or 72 or 73 or 74 or 75 or 76
78. "behavi?r* problem*".ti,ab.
79. "emotion* problem*".ti,ab.
80. "temperament*".ti,ab.
81. self regulation.ti,ab.
82. attachment.ti,ab.
83. self esteem.ti,ab.
84. self efficacy.ti,ab.
85. "social competen*".ti,ab.
86. "peer relationship*".ti,ab.

87. pro-social behavi?r.ti,ab.
88. hyperactivity.ti,ab.
89. impulsivity.ti,ab.
90. attention* deficit hyperactivity disorder*.ti,ab.
91. aggression.ti,ab.
92. exp mental health/ or exp mental disorders/
93. depression/
94. psychology/ or exp adolescent development/ or exp adult development/ or exp emotional development/ or exp self esteem/ or exp self reliance/
95. exp attention deficit hyperactivity disorder/
96. 78 or 79 or 80 or 81 or 82 or 83 or 84 or 85 or 86 or 87 or 88 or 89 or 90 or 91 or 92 or 93 or 94 or 95
97. 50 or 68 or 77 or 96
98. 40 and 97
99. limit 98 to yr="1995 -Current"

PsycINFO

1. severe acute malnutrition.ti,ab.
2. acute malnutrition.ti,ab.
3. (acute adj2 malnutrition).ti,ab.
4. "sever* acute* maln*".ti,ab.
5. protein-energy malnutrition.ti,ab.
6. protein calorie malnutrition.ti,ab.
7. (protein malnutrition or energy malnutrition or energy-protein malnutrition).ti,ab.
8. "acute* maln*".ti,ab.
9. "kwas?io?kor*".ti,ab.
10. "marasm*".ti,ab.
11. "marasm* kwas?io?kor*".ti,ab.
12. wasting.ti,ab.
13. wasted.ti,ab.
14. (acute adj2 wasting).ti,ab.
15. (severe adj2 wasting).ti,ab.
16. acute undernutrition.ti,ab.
17. (acute adj2 undernutrition).ti,ab.
18. severe undernutrition.ti,ab.
19. (severe adj2 undernutrition).ti,ab.
20. "mid* upper arm circumference".ti,ab.
21. MUAC.ti,ab.
22. "acute* undernourish*".ti,ab.
23. "emaciat*".ti,ab.

24. "acut* maln*".ti,ab.
25. nutritional deficiencies/ or protein deficiency disorders/ or exp failure to thrive/ or exp underweight/
26. exp Kwashiorkor/
27. *protein deficiency disorders/
28. 1 or 2 or 3 or 4 or 5 or 6 or 7 or 8 or 9 or 10 or 11 or 12 or 13 or 14 or 15 or 16 or 17 or 18 or 19 or 20 or 21 or 22 or 23 or 24 or 25 or 26 or 27
29. "child*".ti,ab.
30. "infan*".ti,ab.
31. "pre-school*".ti,ab.
32. "preschool*".ti,ab.
33. "toddler*".ti,ab.
34. 29 or 30 or 31 or 32 or 33
35. 28 and 34
36. "child* developm* ".ti,ab.
37. "child* neurodevelopm*".ti,ab.
38. "neurodevelopm*".ti,ab.
39. (socio-emotion* develop* or socioemotion* develop*).ti,ab.
40. "social developm*".ti,ab.
41. "emotion* developm*".ti,ab.
42. (sensorymotor developm* or sensory-motor developm*).ti,ab.
43. "motor developm*".ti,ab.
44. "motor neurodevelopm*".ti,ab.
45. psychomotor developm*.ti,ab.
46. exp infant development/ or exp early childhood development/
47. *childhood development/ or exp early childhood development/ or exp motor development/ or exp psychological development/ or exp psychomotor development/
48. exp emotional development/ or exp psychological development/
49. 36 or 37 or 38 or 39 or 40 or 41 or 42 or 43 or 44 or 45 or 46 or 47 or 48
50. cognition.ti,ab.
51. "cogniti* performance".ti,ab.
52. intelligence.ti,ab.
53. IQ.ti,ab.
54. "executive function*".ti,ab.
55. reasoning.ti,ab.
56. language.ti,ab.
57. attention.ti,ab.
58. memory.ti,ab.
59. learning.ti,ab.
60. early learning.ti,ab.
61. information processing.ti,ab.

62. literacy.ti,ab.
63. reading.ti,ab.
64. math.ti,ab.
65. *cognitive processes/ or exp cognitive assessment/ or exp learning/ or exp memory/ or exp neurocognition/
66. *learning/ or adult learning/ or problem based learning/ or school learning/ or skill learning/ or verbal learning/
67. exp Fine Motor Skill Learning/ or exp Learning Disabilities/ or exp Learning Disorders/ or exp Gross Motor Skill Learning/
68. *language disorders/ or *specific language impairment/ or exp language delay/ or exp language development/
69. *cognitive development/ or exp brain development/ or exp speech development/
70. *cognition/ or exp cognitive impairment/
71. *intelligence/ or exp intellectual development/ or exp intelligence quotient/
72. 50 or 51 or 52 or 53 or 54 or 55 or 56 or 57 or 58 or 59 or 60 or 61 or 62 or 63 or 64 or 65 or 66 or 67 or 68 or 69 or 70 or 71
73. education.ti,ab.
74. "school* achievement".ti,ab.
75. "school* performance".ti,ab.
76. school retention.ti,ab.
77. academic.ti,ab.
78. pre-academic.ti,ab.
79. schooling.ti,ab.
80. *education/
81. education/ or exp academic achievement/ or exp school attendance/ or exp school dropouts/ or exp school learning/
82. 73 or 74 or 75 or 76 or 77 or 78 or 79 or 80 or 81
83. "behavi?r* problem*".ti,ab.
84. "emotion* problem*".ti,ab.
85. "temperament*".ti,ab.
86. self regulation.ti,ab.
87. attachment.ti,ab.
88. self esteem.ti,ab.
89. self efficacy.ti,ab.
90. "social competen*".ti,ab.
91. "peer relationship*".ti,ab.
92. pro-social behavi?r.ti,ab.
93. hyperactivity.ti,ab.
94. impulsivity.ti,ab.
95. attention* deficit hyperactivity disorder*.ti,ab.
96. aggression.ti,ab.

Kirolos A, *et al. BMJ Global Health* 2022; 7:e009330. doi: 10.1136/bmjgh-2022-009330

97. *mental health/
98. exp behavior problems/ or exp behavior disorders/ or exp conduct disorder/
99. exp anxiety disorders/
100. exp hyperkinesis/ or exp attention deficit disorder with hyperactivity/
101. *deprivation/ or exp psychological stress/ or exp stress/
102. *affective disorders/ or *major depression/
103. 83 or 84 or 85 or 86 or 87 or 88 or 89 or 90 or 91 or 92 or 93 or 94 or 95 or 96 or 97 or 98 or 99 or 100 or 101 or 102
104. 49 or 72 or 82 or 103
105. 35 and 104
106. limit 105 to (human and yr="1995 –Current)

Kirolos A, *et al. BMJ Global Health* 2022; 7:e009330. doi: 10.1136/bmjgh-2022-009330

Supplementary Table 1 – Study quality scoring

Study (Year published)	External Validity	Internal Validity	Overall Study Quality
**Chen *et al* (2021)**	++	++	++
**Mwene-Batu *et al* (2020)**	++	+	+
**Asiki *et al* (2019)**	++	++	++
**Dwivedi *et al* (2018)**	+	+	+
**Hock *et al* (2018)**	++	+	+
**Kang *et al* (2018)**	++	+	+
**Lelijiveld *et al* (2018)**	++	++	++
**Abessa *et al* (2017)**	++	++	++
**Sudfeld *et al* (2015)**	++	++	++
**De Grandis *et al* (2014)**	+	-	-
**Waber (1) *et al* (2014)**	++	++	++
**Waber (2) *et al* (2014)**	++	++	++
**Malhi *et al* (2013)**	+	-	-
**Galler *et al* (2013)**	++	+	+
**Bogale *et al* (2013)**	++	++	++
**Galler (1) *et al* (2012)**	++	+	+
**Galler (2) *et al* (2012)**	++	+	+
**Warsito *et al* (2012)**	++	-	-
**Nassar *et al* (2012)**	-	-	-
**Waber *et al* (2011)**	++	+	+
**Galler *et al* (2011)**	++	++	++
**Galler *et al* (2010)**	++	++	++
**Baker-Henningham *et al* (2009)**	++	++	++
**El-Khayat *et al* (2007)**	+	+	+
**Liu *et al* (2004)**	++	++	++
**Liu *et al* (2003)**	++	++	++
**Drewett *et al* (2001)**	++	++	++
**Vazir *et al* (1998)**	++	+	+
**Perales *et al* (1996)**	+	+	+
**Kaul *et al* (1995)**	+	+	+

++ = very good

+ = adequate

- = poor

Kirolos A, *et al. BMJ Global Health* 2022; 7:e009330. doi: 10.1136/bmjgh-2022-009330

Supplementary Table 2 – Results from studies assessing neurodevelopment in children with malnutrition compared to controls

Study (quality)	Neurodevelopment tool/measurement	Summary of results
**Dwivedi *et al* (2018)**<br><br>**(+)**	DASII (Indian modification of BSID)	Controls n=101 / Cases n=102<br>**Mental age:**<br>10.4 (SE 0.5) / 8.2 (SE 0.4)<br><br>**Mental development quotient:**<br>83.5 (SE 1.0) / 62.1 (SE 0.6)<br><br>**Motor age:**<br>10.1 (SE 0.5) / 7.9 (SE 0.4)<br><br>**Motor development quotient:**<br>80.0 (SE 1.0) / 59.0 (SE 0.7)<br><br>**Adjusted analysis:**<br>(unadjusted analysis)<br><br>**Overall findings:**<br>Significant developmental delay in children with severe malnutrition compared to controls unadjusted for confounding variables.
**Kang *et al* 2018**<br><br>**(+)**	MICS ECDI	**Learning-cognition development (odds of cases being on track compared to controls):**<br>Country / Unadjusted Odds Ratio / Adjusted Odds Ratio<br>Bangladesh / 1.27 [95% CI 0.92, 1.75] / 1.27 [95% CI 0.90, 1.79]<br>Bhutan / 1.60 [95% CI 0.32, 7.85] / 1.93 [95% CI 0.39, 9.48]<br>Nepal / 1.09 [95% CI 0.66, 1.79] / 1.52 [95% CI 0.90, 2.58]<br>Punjab / 0.63 [95% CI 0.50, 0.79] / 0.67 [95% CI 0.53, 0.86]<br>Sindh / 0.69 [95% CI 0.53, 0.89] / 0.76 [95% CI 0.59, 0.97]

		All 0.98 [95% CI 0.70, 1.37]<br><br>**Socio-emotional development (odds of cases being on track compared to controls):**<br>Country / Unadjusted Odds Ratio / Adjusted Odds Ratio<br>Bangladesh 1.14 [95% CI 0.92, 1.42] 1.10 [95% CI 0.40, 1.88]<br>Bhutan 1.08 [95% CI 0.58, 2.01] 1.08 [95% CI 0.58, 2.03]<br>Nepal 2.40 [95% CI 1.25, 4.58] 2.54 [95% CI 1.33, 4.85]<br>Punjab 0.86 [95% CI 0.73, 1.02] 0.88 [95% CI 0.74, 1.04]<br>Sindh 0.92 [95% CI 0.74, 1.14] 0.94 [95% CI 0.76, 1.15]<br>All 1.07 [95% CI 0.86, 1.33]<br><br>**Adjusted analysis:** (results adjusted for rural/urban, wealth quintile, household size, household head, water source, toilet facility, maternal education, child age and sex, diarrhoea/cough in past 2 weeks, study design effect, early childhood education, support for learning, availability of children's books and playthings, inadequate care)<br><br>**Sample size:**<br>n = 31037, wasting prevalence = between 4.1-12.8% across study sites<br><br>**Overall findings:**<br>Mixed results between study sites with no significant difference between cases and controls including when adjusted for confounding variables
**Abessa *et al* (2017)**<br><br>**(++)**	Denver II<br><br>ASQ: social-emotional score	**Denver II score (incidence rate ratio in cases compared to controls):**<br>Fine motor IRR 0.76 [95% CI 0.71, 0.8]<br>Gross motor IRR 0.73 [95% CI 0.71, 0.74]<br>Language IRR 0.98 [95% CI 0.86, 1.11]<br>Personal-social IRR 0.7 [95% CI 0.65, 0.75]<br><br>**Adjusted analysis:**<br>(results adjusted for significant variables from age, sex, maternal religion)<br><br>**Sample size:**

		Cases n=310, Controls n=310<br><br>**Overall Findings:**<br>Children with malnutrition performed worse on personal-social, fine motor, and gross motor skills compared to age and sex-matched controls adjusted for confounding variables. Motor skills are the most and personal social the least affected domains assessed on the Denver II Jimma.
**Sudfeld *et al* (2015)**<br><br>**(++)**	BSID-III	**BSID-II score (association between score and malnutrition):**<br>Cognitive -0.63 [95% CI -0.97, -0.29]<br>Communication -0.32 [95% CI -0.64, -0.01]<br>Motor -0.54 [95% CI -0.86, -0.23]<br><br>**Adjusted analysis:**<br>(results adjusted for height-for-age, stimulation category, sex, age, assessor, maternal education, wealth quintile, randomized regimen)<br><br>**Sample size:**<br>Cases n=47, Controls n=989<br><br>**Overall Findings:**<br>Children experiencing wasting had across domain deficits compared to controls adjusted for confounding variables.
**Malhi *et al* 2013**<br><br>**(-)**	Indian Development Inventory	Controls n=20 / Cases n=44 / p value<br><br>**Inventory domain:**<br>Social score 104.64 (SD 17.11) 101.30 (SD 17.31) 0.036<br>Adaptive score 105.36 (SD 9.71) 105.25 (SD 8.33) 0.239<br>Motor score 112 (SD 14.5) 108.85 (SD 10.93) 0.074<br>Communication score 97.82 (SD 14.34) 94.50 (SD 8.61) 0.047<br>Cognitive score 92.82 (SD 13.92) 90.1 (SD 9.79) 0.080<br>Total score 100.86 (SD 18.15) 95 (SD 13.51) 0.007

		**Adjusted analysis:**<br>Multivariate regression revealed that 26.7% of variance was accounted by household income and stunting, indicating results were no longer significant<br><br>**Overall Findings:**<br>Children with wasting compared to controls did not have significantly lower domain scores adjusted for confounding variables.
**El-Khayat *et al* 2007**<br><br>**(+)**	BSID-II	Controls n=15 / Cases n=42 (before rehab) / Cases (after rehab)<br>Mental Development Index: 97 (SD 10.92) / 76.3 (SD 8.78) / 88.19 (SD 8.93)<br>Psychomotor Development Index: 97 (SD 12.38) / 72.76 (SD 11.27) / 87.39 (SD 8.85)<br><br>**Adjusted analysis:**<br>(unadjusted analysis)<br><br>**Overall Findings:**<br>Cases had lower scores at the start of the study compared to controls unadjusted for confounding variables. Scores were improved after 8 weeks of nutritional rehabilitation but were still significantly lower when compared to controls unadjusted for confounding variables.
**Drewett *et al* 2001**<br><br>**(++)**	BSID	Controls n=100 / Cases n=27 Early Malnutrition at 4 months / Cases n=70 Late Malnutrition at 12 months<br>**BSID (score at 2 years):**<br>Psychomotor score: 10.2 (SD3.7) / 6.6 (SD 4.2) / 8.5 (SD 4.3)<br>Mental score: 28.9 (SD5.8) / 22.6 (SD 6.2) / 26.6 (SD 6.1)<br><br>**Adjusted analysis:**<br>Weight at 2 years associated with BSID outcome, but early and late growth faltering before 1 year not independently associated with BSID scores after adjusting for weight at 2 years and other covariates (sex, mother's education, possessions).<br><br>**Overall Findings:**<br>Significantly lower psychomotor and mental scores at 2 years in early growth falterers and late growth

		falterers compared to controls. Difference is accounted for by differences in weight at 2 years when adjusted for in analysis.
**Vazir *et al* 1998**<br><br>**(+)**	ICMR Psychosocial Developmental Screening Test	**ICMR Psychosocial Development Screening Test Results:**<br>Well nourished children attained developmental milestones at a younger age in the five areas of development compared to undernourished children.<br><br>**Adjusted analysis:**<br>(unadjusted analysis)<br><br>**Sample size:**<br>Cases n=1456, Controls n=2212<br><br>**Overall Findings:**<br>Malnourished children attained developmental milestones at a later age unadjusted for confounders.
**Kaul *et al* 1995**<br><br>**(+)**	BSID	Controls n=102 / Cases n=50<br>**BSID (mental quotient):**<br>Male (0-6 months): 98.80 (SD 11.94) / 57.60 (SD 6.77)<br>Female (0-6 months): 97.28 (SD 10.22) / 59.85 (SD 11.94)<br>Male (6-12 months): 100.86 (SD 4.39) / 79.93 (SD 5.57)<br>Female (6-12 months): 103.68 (SD 4.31) / 75.15 (SD 3.39)<br><br>**Adjusted analysis:**<br>(unadjusted analysis)<br><br>**Overall Findings:**<br>Children with moderate malnutrition had a lower mental quotient than controls unadjusted for confounding variables.

Kirolos A, *et al. BMJ Global Health* 2022; 7:e009330. doi: 10.1136/bmjgh-2022-009330

95% CI – 95% confidence intervals, BSID – Bayley Scales of Infant Development, DASII – Developmental Assessment Scale of Indian Infants, ICMR – Indian Council Medical Research Psychosocial Developmental Screening Test, IDI – Indian Developmental Inventory, IRR – incidence rate ratio, MDAT – Malawi Developmental Assessment Tool, MICS ECDI – Multiple Indicator Cluster Survey Early Child Development Index, SD – Standard Deviation, SE – standard error

Kirolos A, *et al. BMJ Global Health* 2022; 7:e009330. doi: 10.1136/bmjgh-2022-009330

Supplementary Table 3 - Results from studies assessing cognitive outcomes in those exposed to childhood malnutrition compared to controls

Study (quality)	Cognition tool/measurement (outcome age)	Summary of results
**Chen *et al* 2021**<br><br>**(++)**	WPPSI-IV<br><br>(childhood)	Controls n=1270 / Cases n=23<br>**WPPSI-IV:**<br>Working Memory Index: Controls 90.35 (SD 0.38) [95% CI 89.60, 91.09]; Cases 95.09 (SD 2.67) [95% CI 89.86, 100.32]<br><br>Verbal Comprehension Index: Controls 86.00 (SD 0.36) [95% CI 85.30, 86.70]; Cases 87.26 (SD 3.29) [95% CI 80.80, 93.72]<br>**Adjusted analysis:**<br>Wasting was not statistically associated with any developmental outcome variables after adjusting for vector of child, parent, caregiver and household-level characteristics.<br><br>**Overall findings:**<br>Wasting was not associated with impaired cognition after adjusting for confounding variables
**Mwene-Batu *et al* 2020**<br><br>**(+)**	MMSE<br>Academic performance<br><br>(adulthood)	Controls n=407 / Cases n=524 / p value<br>**MMSE:**<br>Mean score: 25.6 (SD 2.6) / 27.8 (SD 2.2) / 0.001<br>Normal score (%): 78.0 / 90.1 / <0.001<br><br>**Academic performance (based on self-report):**<br>Low (%): 15.2 / 23.8<br>Average (%): 49.0 / 45.1<br>High (%): 35.8 / 31.0 / 0.014<br><br>**Adjusted analysis:** (unadjusted analysis)<br><br>**Overall findings:**

		Malnutrition survivors had a significantly lower probability of attaining a high education level, reported worse academic performance and a significantly lower mean cognition score unadjusted for confounding variables.
**Asiki *et al* 2019**<br><br>**(++)**	Academic achievement<br><br>(adolescence)	Controls n=464 / Children with ongoing wasting n=12 / Children recovered from wasting n=145<br>**School years achieved (parental report):**<br>9.2 years (SD 11) / 6.2 (SD 2.5) / 7.8 (SD 3.8)<br><br>**Adjusted correlation coefficient:**<br>-1.91 [95% CI -4.51, 0.68] / - 2.05 [95% CI -3.30, -0.79]<br><br>**Adjusted analysis:** (adjusted for age group, sex, education, tribe, wall of house type, reported alcohol consumption)<br><br>**Overall findings:**<br>Wasting was associated with fewer schooling years regardless of recovery from undernutrition adjusted for confounding variables.
**Lelijveld *et al* 2018**<br><br>**(++)**	Academic achievement<br>CANTAB<br><br>(adolescence)	Controls n=178 / Cases n=315<br>**School grade achieved (parental report):**<br>3.1 (SD 1.6) / 2.5 (SD 1.3)<br><br>**Logistic regression comparing school grade in groups:**<br>OR 0.5 [95% CI 0.4, 0.7]<br>Adjusted OR 0.54 [95% CI 0.35, 0.81]<br><br>**Adjusted analysis:** (adjusted for h/a z-score, age, sex, HIV, socioeconomic status)<br><br>**CANTAB cognition test scores:**<br>Malnutrition survivors had poorer scores in all CANTAB cognitive tests.<br><br>**Adjusted analysis:** Adjusting for HIV and socio-economic status diminished statistically significant

		differences in CANTAB test scores.<br><br>**Overall findings:**<br>Malnutrition survivors were significantly more likely to be in a lower grade at school than controls adjusted for confounding variables. Malnutrition survivors had poorer cognition scores than controls, but differences were not statistically significant when adjusted for confounding variables.
**Bogale *et al* 2013**<br><br>**(++)**	Raven's CPM, KABC-II<br><br>(childhood)	Controls n=88 / Cases n=12 / p value<br>**Cognitive scores:**<br>Visual processing: 17.0 (SD 3.9) / 12.8 (SD 2.4) / 0.003<br>Triangles: 4.3 (SD 2.4) / 1.7 (SD 1.0) / 0.001<br>Conceptual thinking: 4.7 (SD 1.8) / 3.3 (SD 1.3) / 0.017<br>Pattern reasoning: 8.1 (SD 1.8) / 7.9 (SD 1.7) / 0.726<br>Short-term memory: 21.7 (SD 5.6) / 16.2 (SD 5.1) / 0.006<br>Word order: 6.5 (SD 3.0) / 4.8 (SD 2.0) / 0.1<br>Number recall: 7.1 (SD 2.2) / 5.4 (SD 2.5) / 0.025<br>Hand movement: 8.1 (SD 2.1) / 5.9 (SD 1.1) / 0.004<br><br>**Adjusted analysis:**<br>When results adjusted via stepwise regression, visual processing and short-term memory remained significantly correlated when adjusted for maternal education, family size, and household roof type.<br><br>**Overall findings:**<br>Scores on short-term memory and visual processing were significantly poorer in cases compared to controls when adjusted for confounding variables.
**Warsito *et al* 2012**<br><br>**(-)**	Indonesian Department of National Education – child development instrument<br><br>(childhood)	Controls n=48 / Wasting n=3 / Severe Wasting n=2<br>**Child development score:**<br>Percentage score: 68.7 / 73.9 / 73.8<br><br>**Adjusted analysis:**<br>(results unadjusted)

		**Overall findings:**<br>Those with wasting in this study had better development scores than those without wasting. The study is limited by small case numbers.
**Nassar *et al* 2012**<br><br>**(-)**	Stanford-Binet-Intelligence-Scale<br>Arabic-Language-Test<br><br>(childhood)	Controls n=30 / Cases n=33 / p value<br>**Stanford-Binet-Intelligence-Scale (mean):**<br>IQ score 92.07 (SD 11.52) 80.52 (SD 13.65) 0.001<br>Mental age (months) 50.50 (SD 13.40) 42.58 (SD 8.66) 0.017<br><br>**Arabic Language Test (mean):**<br>Receptive language age (months) 51.67 (SD 13.98) 40.09 (SD 17.36) 0.005<br>Expressive language age (months) 51.67 (SD 14.23) 40.76 (SD 16.74) 0.003<br>Total language age (months) 51.40 (SD 14.15) 40.00 (SD 17.66) 0.007<br><br>**Adjusted analysis:**<br>(results unadjusted)<br><br>**Overall findings:**<br>Those with malnutrition had poorer scores for both tests than age- and sex-matched controls unadjusted for confounding variables.
**Liu *et al* 2003**<br><br>**(++)**	Bohem Test<br>WISC<br>Academic Tests<br>Holborn-Reading Scale<br>Trail Making Test<br><br>(adolescence)	Control n=837 / Cases n=253 / p value<br>**Age 3y:**<br>Verbal IQ 100.92 (SD 15.04) 96.93 (SD 14.36) 0.001<br>Spatial IQ 100.34 (SD 15.21) 98.95 (SD 13.52) 0.18<br>Full-Scale IQ 100.92 (SD 15.04) 96.93 (SD 14.36) 0.001<br><br>**Age 11y:**<br><br>Verbal IQ 100.90 (SD 14.41) 96.16 (SD 15.40) 0.001<br>Spatial IQ 100.75 (SD 14.55) 96.23 (SD 15.41) 0.001<br>Full-Scale IQ 100.88 (SD 14.43) 95.83 (SD 15.49) 0.001<br>Reading 95.11 (SD 55.30) 76.81 (SD 56.58) 0.001

		School performance 10.33 (SD 7.07) 7.63 (SD 6.48) 0.001<br>Trails A -0.049 (SD 0.95) 0.24 (SD 1.10) 0.001<br>Trails B -0.023 (SD 0.98) 0.20 (SD 1.07) 0.004<br><br>**Adjusted analysis:**<br>Differences remain after controlling for psychosocial adversity.<br><br>**Overall Findings:**<br>Malnourished children had poorer cognition and school/academic performance at both ages after controlling for psychosocial adversity.
**Perales *et al* 1996**<br><br>**(+)**	Continuous Performance Task<br>Anstey Domino Test<br><br>(childhood)	Controls n=40 Cases n=40 p value<br>**Short-term Memory**:<br>Number-of-digits score 4.5 (SD 0.87) 3.8 (SD 0.88) <0.05<br><br>**Problem-Solving:**<br>Correct score 4.7 (SD 2.1) 3.4 (SD 1.4) <0.05<br><br>**Attention**:<br>No differences in correct answers between groups. Lower performance in cases response time for correct answers (p<0.02).<br><br>**Adjusted analysis:**<br>(unadjusted analysis)<br><br>**Overall Findings:**<br>Cases had lower memory scores and problem solving scores unadjusted for confounding variables. Cases had worse performance than controls for response times in the attention test unadjusted for confounding variables.
Barbados Nutrition Study		
**Waber (1) *et al* 2014***	Academic achievement, WASI	**WRAT-III academic achievement score (adjusted odds ratio):**<br>Reading OR 3.44 [95% CI 1.88–6.30]

**(++)**	(adulthood)	Spelling OR 6.10 [95% CI 3.34–11.16]<br>Calculation OR 6.23 [95% CI 3.15–12.32]<br><br>**<u>WASI score (adjusted odds ratio):</u>**<br>IQ OR 9.18 [95% CI 3.50, 24.13]<br><br>**<u>Adjusted analysis:</u>**<br>(Results adjusted for childhood standard of living)<br><br>**<u>Sample size:</u>**<br>Cases n=77, Controls n=59<br><br>**<u>Overall findings:</u>**<br>Academic skills and IQ were lower in the malnourished group compared to controls after adjusting for childhood standard of living.
**Waber (2) *et al* 2014***<br><br>**(++)**	WAIS-III, D-KEFS, WRAML-II, Wisconsin card sorting, Metacognitive index<br><br>(adulthood)	Controls n=59 / Cases n=77<br><br>**<u>Metacognitive index (General executive composite)</u>**:<br>Mean score 41.81 (SD 6.05) / 47.36 (SD 9.33)<br><br>Adjusted Mean difference<br>3.70 (p<0.05)<br><br>**<u>WRAML-2:</u>**<br>Scaled score 8.49 (SD 1.81) / 7.45 (SD 2.28)<br><br>Adjusted Mean difference<br>-0.42 (p>0.05)<br><br>**<u>Wisconsin card sorting:</u>**<br>Total errors score 94.56 (SD 12.70) / 80.67 (SD 12.53)

		Adjusted Mean difference<br>-9.74 (p<0.001)<br><br>**Adjusted analysis**<br>(results adjusted for childhood standard of living and IQ)<br><br>Significant differences persisted for the WAIS Digit Symbol and Letter-Number, D-KEFS Trails (Numbers) , Verbal Fluency (Letters) and Rey-Osterrieth Complex Figure after adjusting for covariates<br><br>**Overall findings:**<br>Malnutrition during infancy is associated with impaired IQ in adulthood. In models adjusted only for childhood standard of living, the malnutrition group performed more poorly on nearly all measures. In the IQ-adjusted models, significant differences persisted for the WAIS Digit Symbol and Letter-Number, D-KEFS Trails (Numbers) and Verbal Fluency (Letters), Rey-Osterrieth Complex Figure, and Wisconsin Card Sorting Test.
**Waber *et al* 2011**<br><br>**(+)**	WISC, Common entrance examination (local school test)<br><br>(adolescence)	Controls n=60 / Malnutrition n=56 / p value<br>**WISC:**<br>IQ: 104.92 (SD 10.54) / 92.54 (SD 13.86) / <0.0001<br><br>**Common Entrance Exam:**<br>Score: 210.42 (SD 22.13) / 184.10 (29.27) / <0.0001<br><br>**Adjusted analysis:**<br>Early malnutrition predicted cognitive function and exam score in childhood after adjusting for childhood standard of living.<br><br>**Overall findings:**<br>Malnutrition predicted common entrance exam score and cognitive functioning after adjusting for childhood standard of living.

*Studies are drawn from the same Barbados Nutrition Study

95% CI – 95% Confidence Intervals, CANTAB – Cambridge Neuropsychological Test Automated Battery, D-KEFS – Delis-Kaplan Executive Function System, MMSE – Mini-Mental State Exam, OR – Odds Ratio, SD – Standard Deviation, WAIS-III – Wechsler Adult Intelligence Scale-III, WASI – Adult IQ: Weschler Abbreviated Scale of Intelligence – Vocabulary and Matrix Reasoning subsets, WISC – Weschler Intelligence Scale, WPPSI-IV – Wechsler Preschool and Primary Scale of Intelligence Fourth Edition, WRAML-II – Wide Range Assessment of Memory and Learning-II, WRAT-III – Academic achievement: Wide Range Achievement Test-III – Reading Spelling and Calculation subsets

Kirolos A, *et al. BMJ Global Health* 2022; 7:e009330. doi: 10.1136/bmjgh-2022-009330

Supplementary Table 4 - Results from studies assessing mental health outcomes in those exposed to childhood malnutrition compared to controls

**Study (quality)**	**Mental health tool/measurement (outcome age)**	**Summary of results**
**Chen *et al* 2021**<br><br>**(++)**	SDQ<br><br>(childhood)	Controls n=1270 / Wasting n=23<br>**SDQ:**<br>Prosocial score: 6.83 (SD 0.06) [95% CI 6.71, 6.95] / 6.70 (SD 0.42) [95% CI 5.86, 7.53]<br>Total difficulties score: 12.35 (SD 0.13) [95% CI 12.10, 12.61] / 12.09 (SD 1.09) [95% CI 9.95, 14.22]<br>**Adjusted analysis:**<br>Wasting was not statistically associated with any developmental outcome variables after adjusting for vector of child, parent, caregiver and household-level characteristics.<br><br>**Overall findings:**<br>Wasting was not associated with a difference in behaviour after adjusting for confounding variables.
**Mwene-Batu *et al* 2020**<br><br>**(+)**	Rosenberg self-esteem scale, WHODAS<br><br>(adulthood)	Controls n=407 / Cases n=524 / p value<br>**Self-esteem:**<br>Low (%): 12.1 / 20.5<br>Average (%): 78.5 / 72.6<br>High (%): 9.4 / 6.9 / 0.003<br><br>**Social related disability:**<br>No disability (%): 91.0 / 94.2<br>Moderate disability (%): 8.8 / 5.0<br>Severe disability (%): 0.3 / 0.8 / 0.03<br><br>**Adjusted analysis:**

Kirolos A, *et al. BMJ Global Health* 2022; 7:e009330. doi: 10.1136/bmjgh-2022-009330

		(unadjusted results)<br><br>**Overall findings:**<br>Cases had significantly less disability in terms of social relationships than the community controls but had statistically significant lower self-esteem than the controls unadjusted for confounding variables.
**Abessa *et al* (2017)**<br><br>**(++)**	ASQ: social-emotional score<br><br>(childhood)	**ASQ: Socio-emotional score (incidence rate ratio in cases compared to controls):**<br>Socioemotional IRR 3.1 [95% CI 2.24, 4.26]<br><br>**Adjusted analysis:**<br>(results adjusted for significant variables from age, sex, maternal religion)<br><br>**Sample size:**<br>Cases n=310, Controls n=310<br><br>**Overall findings:**<br>Children with malnutrition performed worse on socio-emotional competences compared to age and sex-matched non-malnourished children adjusted for confounding variables.
**De Grandis *et al* 2014**<br><br>**(-)**	Paediatric quality of life inventory<br><br>(childhood)	Controls n=28 / Malnutrition n=25 / p value<br>**Peds Q:**<br>Global score 89.18 (SD 1.84) 80.82 (SD 1.94) p<0.0001<br>Health/physical dimension 94.75 (SD 1.87) 87.75 (SD 3.37) p<0.0001<br>Psychosocial health 86.57 (SD 1.42) 77.77 (SD 2.90) p<0.0001<br>Emotional dimension 78.75 (SD 2.96) 67.80 (SD 4.40) p<0.0001<br>Social dimension 95.71 (SD 1.52) 88.80 (SD 3.05) p<0.0001<br>School dimension 74.58 (SD 3.80) 85.00 (SD 3.51) p<0.0001<br><br>**Adjusted analysis:**<br>(unadjusted analysis)<br><br>**Overall findings:**<br>Those with a history of early severe malnutrition, showed significantly lower quality of life scores compared to

		controls unadjusted for confounding variables.
**Baker-Henningham *et al* 2009**<br><br>**(++)**	Temperament Questionnaire derived from existing validated instruments (modified by Wachs, Purdue University)<br><br>(childhood)	Controls n=108 / Undernourished n=212 / p value<br>**Temperament questionnaire:**<br>Manageability: 8.67 (SD 3.51) / 9.56 (SD 3.26) / p<0.05<br>Activity: 10.40 (SD 3.00) / 10.59 (SD 2.73) / p>0.05<br>Emotionality: 13.66 (SD 4.74) / 14.80 (SD 4.24) / p<0.05<br>Sociability: 8.15 (SD 3.87) / 9.29 (SD 3.98) / p<0.05<br>Attention: 12.58 (SD 3.31) / 13.53 (SD 3.06) / p<0.05<br>Soothability: 8.23 (SD 2.77) / 8.42 (SD 2.57) / p<0.01<br><br>**Adjusted analysis:**<br>After adjusting for significant covariates (parental schooling and occupation, sanitiation index, asset index, housing index, mother's BMI and home), the undernourished children were less sociable (regression coefficient (B) = -0.96 [95% CI -0.04, -1.88]), less attentive (B = -0.94 [95% CI -0.19, -1.69], more fearful (B = 1.43 [95% CI 2.44, 0.42]), and had more negative emotionality (B = -1.08 [95% CI -2.16, 0.006]).<br><br>**Overall findings:**<br>There were significant differences in temperament traits between undernourished and better-nourished groups after adjusting for confounding variables.
**Liu *et al* (2004)**	Child behaviour questionnaire at age 8, Child Behaviour Checklist at age 11, Revised Behaviour Checklist at Age 17<br><br>(childhood, adolescence)	**Externalizing behaviour problems at age 8:**<br>Malnourished children had higher overall externalizing behaviour scores (both hyperactivity and aggression) than controls.<br><br>**Adjusted analysis:**<br>Main effect of malnutrition remained significant after controlling for psychosocial adversity but was abolished after controlling for cognitive ability.<br><br>**Externalizing behaviour problems at age 11:**<br>Malnourished children had higher externalising behaviour scores (hyperactivity only) at age 11 than non-malnourished children.

		**Adjusted analysis:**<br>Main effect of malnutrition remained significant after controlling for psychosocial adversity but was abolished after controlling for cognitive ability.<br><br>**Externalizing behaviour problems at age 17:**<br>Malnourished group had significantly higher scores on conduct disorder and motor excess but not attention problems or socialized aggression.<br><br>**Adjusted analysis:**<br>Main effect of malnutrition remained significant after adjusting for psychosocial adversity and cognitive ability.<br><br>**Sample size:**<br>Malnutrition group n=235, Control group n=807<br><br>**Overall Findings:**<br>Children with malnutrition were more aggressive or hyperactive at age 8 years, had more externalizing problems at age 11, and had greater conduct disorder and excessive motor activity at age 17 after adjusting for confounding variables.
Barbados Nutrition Study		
**Hock *et al* 2018***<br><br>**(+)**	SCID-II-PQ, NEO PI-R FFM<br><br>(adulthood)	**SCID-II-PQ (linear regression with malnutrition as predictor of score):**<br>Paranoid personality score 0.6 [95% CI 0.0, 1.2] (p=0.0499)<br><br>**Adjusted analysis:**<br>PD paranoid scale score had unique association with malnutrition after adjusting for maltreatment and childhood standard of living<br><br>**NEO PI-R FFM (linear regression with malnutrition as predictor of score):**<br>Avoidant personality score 1.0 [95% CI 0.2, 1.7] (p=0.009), schizoid personality score 0.7 [95% CI 0.1, 1.4] (p=0.03) and dependant personality score 0.7 [95% CI 0.1, 1.4] (p=0.04)<br><br>**Adjusted analysis:**

		PD avoidant, schizoid and dependant scale scores had unique associations with malnutrition after adjusting for maltreatment and childhood standard of living<br><br>**Sample size:**<br>Malnutrition group n=77, control group n=62<br><br>**Overall findings:**<br>Adults with history of infant malnutrition showed an increase in most SCID-II and NEO FFM PD personality scores compared to those with little or no exposure to either adversity. Few personality disorder score differences were statistically significant however and results differed depending on the assessment tool used.
**Waber (2) *et al* 2014***<br><br>**(++)**	Behavioural Regulation Index score<br><br>(adulthood)	Controls n=59 Malnutrition n=77<br><br>**Behavioural regulation index**:<br>Mean score 43.34 (SD 7.52) 49.51 (SD 11.21)<br><br>Mean difference (adjusted)<br>3.40 (p<0.05)<br><br>**Adjusted analysis**<br>(results adjusted for childhood standard of living and IQ)<br><br>**Overall findings:**<br>Adults with a history of infant malnutrition showed a significantly higher behavioural regulation index score which is indicative of more behavioural problems, with a significant difference in score after adjusting for confounding variables.
**Galler *et al* 2013***<br><br>**(+)**	NEO-PI-R personality inventory<br><br>(adulthood)	**NEO-PI-R score:**<br>At the broad domain or factor level, previously malnourished participants had higher scores on Neuroticism and lower scores on Extraversion, Openness, Agreeableness and Conscientiousness than healthy controls. At the sub-domain or facet level, previously malnourished participants reported more anxiety, vulnerability, shyness and lowered sociability, less intellectual curiosity, greater suspiciousness of others, a more egocentric than altruistic

		orientation, and a lowered sense of efficacy or competence.<br><br>**<u>Adjusted analysis:</u>**<br>(results adjusted for childhood standard of living)<br><br>**<u>Sample size</u>**<br>Malnutrition group n=77, Control group n=57<br><br>**<u>Overall findings:</u>**<br>Childhood malnutrition was associated with higher chance of personality trait scores outside of the average range after adjusting for childhood standard of living.
**Galler (1) *et al* 2012***<br><br>**(+)**	CAARS, CPT<br><br>(adulthood)	Controls n=63 — Malnutrition n=80 — p value<br>**<u>CAARS (mean score):</u>**<br>Inattentive symptoms — 3.6 (SD 0.1) — 3.7 (SD 0.2) — <0.05<br>Hyperactive symptoms — 3.7 (SD 0.1) — 3.7 (SD 0.2) — >0.05<br>DSM-IV ADHD symptoms — 3.6 (SD 0.1) — 3.7 (SD 0.1) — <0.01<br>ADHD index — 3.8 (SD 0.2) — 3.7 (SD 0.1) — >0.05<br><br>Controls n=59 — Malnutrition n=77 — p value<br>**<u>CPT (mean score):</u>**<br>Commission errors — 3.8 (SD 0.2) — 3.9 (SD 0.2) — >0.05<br>Omission errors — 3.9 (SD 0.2) — 4.1 (SD 0.3) — >0.05<br>Reaction time — 4.0 (SD 0.2) — 4.0 (SD 0.2) — >0.05<br><br>**<u>Adjusted analysis:</u>**<br>(results adjusted for childhood standard of living and IQ)<br><br>**<u>Overall findings:</u>**<br>CAARS scores were higher (indicating more attention problems) in the previously malnourished group. There were significant differences in inattention and DSM-IV ADHD symptom scores after adjusting for confounding variables. Differences in CPT scores were attenuated after adjusting for confounding variables.

**Galler (2) *et al* 2012***<br><br>**(+)**	Conduct-problems: Child-Behaviour-Questionnaire, Teacher-Behaviour-Questionnaire<br><br>(adolescence)	Controls n=60 Malnutrition n=56 p value<br>**Child-Behaviour-Questionnaire (coefficient):**<br>Conduct problems -0.27 (SD 0.84) 0.29 (SD 1.18) <0.01<br><br>**Teacher-behaviour-questionnaire:**<br>All four items showed a higher level prevalence of aberrant behavior in the previously malnourished children as compared with the comparison group.<br><br>**Adjusted analysis:**<br>The difference was no-longer-significant with IQ and childhood standard of living included in the model<br><br>**Overall findings:**<br>Self-reported conduct problems were more prevalent among previously malnourished youth. The associated was no longer significant when adjusted for confounding variables.
**Waber *et al* 2011***<br><br>**(+)**	Teacher-Behaviour Questionnaire, School functioning scale Minnesota-General-Adjustment-and Morale-Scale<br><br>(adolescence)	Controls n=60 Malnutrition n=56 p value<br>**Teacher questionnaire:**<br>Attention symptoms -0.36 (SD 0.81) 0.33 (SD 0.96) <0.0001<br><br>**Morale scale:**<br>Depressive symptoms -0.33 (SD 0.93) 0.30 (SD 1.01) 0.001<br><br>**Adjusted analysis:**<br>Association attenuated when IQ and attention problems included in model. Difference in attention symptoms not significant when adjusting childhood standard of living.<br><br>**Overall findings:**<br>Depressive symptoms and inattention were higher in those with malnutrition but there were no significant differences when adjusting for confounding variables.
**Galler *et al* 2011***	Barbados-Child-Behaviour-Scale	Controls n=109 Malnutrition n=107<br>(correlations with mean score)<br>**Peer aggression:**

**(++)**	adolescence	Time point 1 (9-15y) -0.05 (SD 0.96) 0.39 (SD 1.16)<br>Time point 2 (11-17y) -0.34 (SD 0.79) -0.34 (SD 0.80)<br>**Cooperative with adults:**<br>Time point 1 (9-15y) 0.19 (SD 1.11) -0.01 (SD 1.15)<br>Time point 2 (11-17y) 0.15 (SD 0.73) -0.28 (SD 1.00)<br>**Activity level:**<br>Time point 1 (9-15y) 0.32 (SD 1.09) 0.18 (SD 1.03)<br>Time point 2 (11-17y) -0.35 (SD 0.72) -0.29 (SD 0.78)<br>**Executive function deficits:**<br>Time point 1 (9-15y) 0.0 (SD 0.94) 0.24 (SD 1.00)<br>Time point 2 (11-17y) -0.38 (SD 0.82) -0.09 (SD 0.94)<br>**Sleep problems:**<br>Time point 1 (9-15y) -0.45 (SD 1.17) -0.24 (SD 1.08)<br>Time point 2 (11-17y) 0.21 (SD 0.61) 0.43 (SD 0.68)<br><br>**Adjusted analysis:**<br>Cases had increased prevalence of executive function deficits at both time points after adjusting for age, sex, childhood standard of living and maternal depressive symptoms. No significant association with aggression, cooperation, activity and sleep after adjusting for covariates at second time point. Cases had more problem aggression at the first time point than at the second.<br><br>**Overall findings:**<br>Persisting parent-reported executive functioning deficits through adolescence at two time points after adjusting for confounding variables.
**Galler *et al* 2010***<br><br>**(++)**	Minnesota-General-Adjustment-and Morale-Scale<br><br>(adolescence)	Controls n=60 Cases n=56 p value<br>**Morale Scale:**<br>Youth depression score -0.33 (SD 0.98) 0.30 (SD 1.01) <0.001<br><br>**Adjusted analysis:**<br>Adjusting for age, maternal depression symptoms and household standard of living the effect of malnutrition is similar to unadjusted estimates and remains significant.

		**Overall findings:**<br>Early childhood malnutrition contributed independently to depressive symptoms after adjusting for confounding variables.

*Studies are drawn from the same Barbados Nutrition Study

95% CI – 95% Confidence Intervals, CAARS - Attention-domain: Conners-ADHD-Rating-Scales, CPT – Conners-Continuous-Performance Test, NEO PI-R FFM – NEO Personality Inventory-Revised derived Five-Factor Model, SD – Standard Deviation, SDQ – Strengths and Difficulties Questionnaire, SCID-II-PQ – Structured Clinical Interview for DSM-IV Axis II Personality Disorders Personality Questionnaire, WHODAS – World Health Organization Disability Assessment Schedule

Kirolos A, *et al. BMJ Global Health* 2022; 7:e009330. doi: 10.1136/bmjgh-2022-009330
